# Structure-Activity Relationships in Alkoxylated Resorcinarenes: Synthesis, Structural Features, and Bacterial Biofilm-Modulating Properties

**DOI:** 10.3390/molecules30153304

**Published:** 2025-08-07

**Authors:** Mariusz Urbaniak, Łukasz Lechowicz, Barbara Gawdzik, Maciej Hodorowicz, Ewelina Wielgus

**Affiliations:** 1 Institute of Chemistry, Faculty of Natural Sciences, Jan Kochanowski University, Uniwersytecka 7, 25-406 Kielce, Poland; b.gawdzik@ujk.edu.pl; 2 Institute of Biology, Faculty of Natural Sciences, Jan Kochanowski University, Uniwersytecka 7, 25-406 Kielce, Poland; lukasz.lechowicz@ujk.edu.pl; 3 Faculty of Chemistry, Jagiellonian University, Gronostajowa 2, 30-387 Kraków, Poland; maciej.hodorowicz@uj.edu.pl; 4 Centre of Molecular and Macromolecular Studies, Polish Academy of Sciences, Sienkiewicza 112, 90-363 Lodz, Poland; ewelina.wielgus@cbmm.lodz.pl

**Keywords:** resorcinarene, alkoxylation, biofilm formation, bacterial stress response

## Abstract

In this study, a series of novel alkoxylated resorcinarenes were synthesized using secondary and tertiary alcohols under mild catalytic conditions involving iminodiacetic acid. Structural characterization, including single-crystal X-ray diffraction, confirmed the successful incorporation of branched alkyl chains and highlighted the influence of substitution patterns on molecular packing. Notably, detailed mass spectrometric analysis revealed that, under specific conditions, the reaction pathway may shift toward the formation of defined oligomeric species with supramolecular characteristics—an observation that adds a new dimension to the synthetic potential of this system. To complement the chemical analysis, selected derivatives were evaluated for biological activity, focusing on bacterial growth and biofilm formation. Using four clinically relevant strains (*Staphylococcus aureus*, *Escherichia coli*, *Pseudomonas aeruginosa*, and *Bacillus subtilis*), we assessed both planktonic proliferation (OD_600_) and biofilm biomass (crystal violet assay). Compound **2c** (2-pentanol derivative) consistently promoted biofilm formation, particularly in *S. aureus* and *B. subtilis*, while having limited cytotoxic effects. In contrast, compound **2e** and the DMSO control exhibited minimal impact on biofilm development. The results suggest that specific structural features of the alkoxy chains may modulate microbial responses, potentially via membrane stress or quorum sensing interference. This work highlights the dual relevance of alkoxylated resorcinarenes as both supramolecular building blocks and modulators of microbial behavior.

## 1. Introduction

Resorcinarenes are a class of macrocyclic compounds formed through the condensation of resorcinol and formaldehyde. They have gained significant attention due to their unique structural features, including a well-defined macrocyclic cavity, conformational flexibility, and the ability to form host-guest complexes. These characteristics make resorcinarenes particularly useful in catalysis, nanotechnology, and supramolecular chemistry [1,2,3]. More recently, resorcinarenes have also found promising pharmaceutical applications as bioactive compounds [4]. However, the majority of studies focus on their use in drug delivery systems (DDS), enabling targeted transport of therapeutic agents to specific tissues or cells. Such targeted delivery increases therapeutic efficacy and minimizes undesirable side effects [5,6,7]. By introducing specific functional groups into the resorcinarene structure, it is possible to bind biomolecules selectively, thereby improving DDS efficiency or developing novel biomedical applications such as targeted therapy or diagnostic systems [8].

Secondary and tertiary alcohols are known to affect the structure, fluidity, and permeability of cellular membranes. Some of these alcohols can interact with lipids, altering membrane fluidity, which in turn influences the transport of molecules across the membrane [9,10,11]. Others may act as ligands for membrane proteins, modulating receptor activity and affecting biological function.

In this study, we present a method for introducing branched alkoxy substituents into the resorcinarene framework using a Mannich-type reaction catalyzed by iminodiacetic acid and secondary and tertiary alcohols. This represents a promising direction in the functionalization of resorcinarenes. The introduction of chiral, branched alkoxy groups may significantly enhance interactions with biological membranes, offering new opportunities in nanomedicine and biomedicine, particularly in the design of next-generation DDS or cell pathway-targeting systems.

Recent reviews have highlighted the growing interest in the biomedical applications of calixarenes and their analogues, including resorcinarenes, particularly in the context of antimicrobial and antibiofilm activity [12,13]. These macrocyclic hosts can be functionalized to interact selectively with microbial membranes, modulate quorum sensing pathways, or deliver therapeutic agents in a targeted manner. Although most studies to date have focused on calix[n]arenes, resorcinarenes are increasingly recognized as versatile platforms for supramolecular engineering, due to their ease of derivatization and potential for forming higher-order assemblies [14]. Notably, recent work has demonstrated that resorcinarene derivatives can inhibit bacterial adhesion or promote biofilm disruption, depending on their functional groups and surface presentation [12]. These advances underscore the relevance of expanding the chemical space of resorcinarenes for biological applications—including the antibiofilm focus of this study.

## 2. Results

### 2.1. Synthesis

A series of novel alkoxylated resorcinarenes were synthesized using a Mannich-type reaction. These compounds have not been previously described, and full spectral characterization is provided in the Supplementary Information. In previous studies, we described a method for introducing linear alkoxy groups to the resorcinarene structure via an iminodiacetic acid-catalyzed Mannich reaction with primary alcohols [15,16,17,18]. While this method yielded methoxy derivatives with up to 88% yield, the efficiency dropped significantly with longer alkyl chains (e.g., 36% yield with n-hexanol), and no derivatives with branched or extended alkyl groups could be obtained.

**Figure 1:**
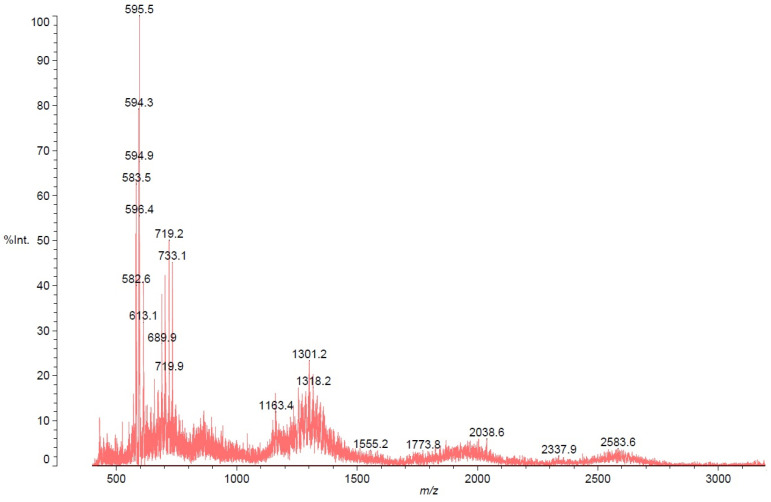
MALDI-TOF mass spectrum showing oligomeric species formed via methylene-bridged resorcinarenes (*m*/*z* 628–2543).

MALDI-TOF analysis revealed that this loss in yield was due to a competitive oligomerization reaction, leading to the formation of poorly soluble products. These products either caused turbidity in the reaction mixture or precipitated as a cream-colored solid, depending on the reaction conditions. The mass spectra indicated the formation of dimers, trimers, and tetramers, with signals clustered at *m*/*z* 628, 1245, 1910, and 2543 (Figure 1).

The synthesis of arylmethylene derivatives of resorcinarenes in reactions with activated aromatic rings acting as nucleophiles has also been described previously [19]. This points to oligomerization via methylene bridges, leading to the formation of larger supramolecular assemblies. These oligomers differ in charge and in the number of aminomethylene substituents. The peak patterns clustered around *m*/*z* 628 and 1245 display a band distribution characteristic of polymers, differing by a constant mass interval, suggesting the presence of oligomers with varying degrees of methylation.

To further investigate this process, we analyzed the reaction of resorcinarene (0.3 g) with formaldehyde (0.5 mL) and iminodiacetic acid (0.03 g, 0.4 eq) in acetonitrile, without the addition of alcohols. This simplified setup aimed to facilitate monitoring of the reaction and to isolate products in quantities sufficient for analysis. Reaction progress was monitored by high-resolution ESI-MS at regular intervals from the start of the reaction until turbidity developed and product precipitation was observed.

**Figure 2:**
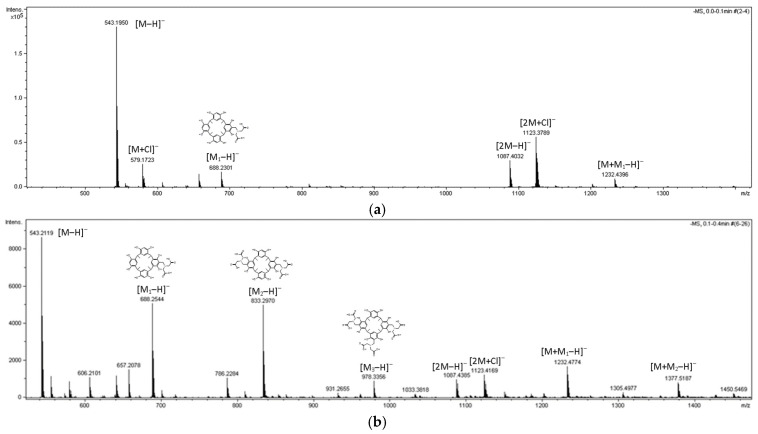
ESI(–)-TOF mass spectrum of the reaction mixture taken at (**a**) 15 min and (**b**) 45 min of the reaction.

The first product, an aminomethylene derivative resulting from the attachment of a single molecule of iminodiacetic acid to the resorcinarene, was detected after approximately 15 min, while the reaction mixture was still clear and the acid was not yet fully dissolved (*m*/*z* 688.2301 [M_1_ − H]^−^; Figure 2a). After 45 min, turbidity developed and additional peaks appeared, corresponding to more highly substituted products. Peaks at *m*/*z* 833.2984 and 978.3343 were assigned to di- and triaminomethylene derivatives [M_2_ − H]^−^ and [M_3_ − H]^−^, respectively (Figure 2b). Although the molar ratio of resorcinarene to iminodiacetic acid was 2.5:1, significant amounts of di- and trisubstituted products were present in the reaction mixture.

The coexistence of partially substituted aminomethylene resorcinarene derivatives is consistent with the proposed reaction mechanism [15]. However, it had previously been assumed that such intermediates were short-lived and that iminodiacetic acid would be rapidly displaced by other nucleophiles present in the reaction medium, such as alcohols or activated aromatic rings. Although the molar ratio of resorcinarene to iminodiacetic acid was 2.5:1, significant amounts of di- and trisubstituted products were present in the reaction mixture. The coexistence of partially substituted aminomethylene resorcinarene derivatives is consistent with the proposed reaction mechanism [15]. However, it had previously been assumed that such intermediates were short-lived and that iminodiacetic acid would be rapidly displaced by other nucleophiles present in the reaction medium, such as alcohols or activated aromatic rings.

Contrary to this assumption, we observed that the addition of alcohol after the onset of turbidity did not lead to the formation of alkoxylated products. At this stage, mono-, di-, and triaminomethylene derivatives were already present in the reaction mixture, and the reaction instead resulted in the precipitation of oligomeric species. In contrast, introducing alcohol at an earlier stage, when the mono-substituted intermediate was predominant, led to the formation of alkoxylated products along with a noticeably reduced amount of precipitate arising from a competing reaction.

**Scheme 1:**
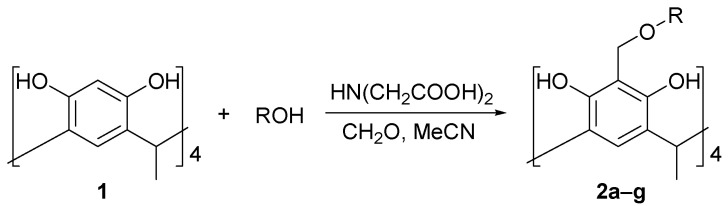
Synthesis of resorcinarene derivatives with branched alkyl groups **2a**–**g**.

These results suggest that the key to achieving selective alkoxylation lies in carefully controlling the concentration of iminodiacetic acid. Optimal yields were obtained when the molar ratio of resorcinarene to iminodiacetic acid was maintained between 8:1 and 5:1. The synthesis required a significant excess of the appropriate secondary or tertiary alcohol and gentle heating for approximately 72 h (Scheme 1). These conditions enabled the synthesis of new derivatives using secondary and tertiary alcohols, with moderate yields comparable to those obtained from primary alcohols. The yields of isolated products after chromatographic purification ranged from 62% to 74% (Table 1). Notably, for secondary alcohols, there was no clear correlation between the length of the aliphatic chain and a decrease in yield. Compared to earlier methods, the use of higher dilution and lower temperature extended reaction time but improved control. Reactions progressed through partially alkoxylated intermediates, readily isolated by chromatography [15]. The notably lower yields observed for tertiary alcohols (e.g., 36% for *tert*-butyl and 29% for *tert*-amyl) highlight the steric limitations of the Mannich-type alkoxylation under the applied conditions.

For longer-chain alcohols, these intermediates occasionally precipitated; the addition of chloroform dissolved such solids, boosting yields of fully substituted products. Reaction times varied slightly depending on the alcohol used. To maximize yields, the reaction was monitored chromatographically to ensure the mixture remained clear and homogeneous, free of precipitation. A key improvement over earlier methods was a significant reduction in the amount of catalyst (to 1/5 equivalent), combined with prolonged reaction time. This approach minimized the formation of polymeric byproducts and favored the selective formation of the monoaminomethylene intermediate, thereby improving the overall efficiency and yield of alkoxylated products.

### 2.2. Crystallographic Studies

**Figure 3:**
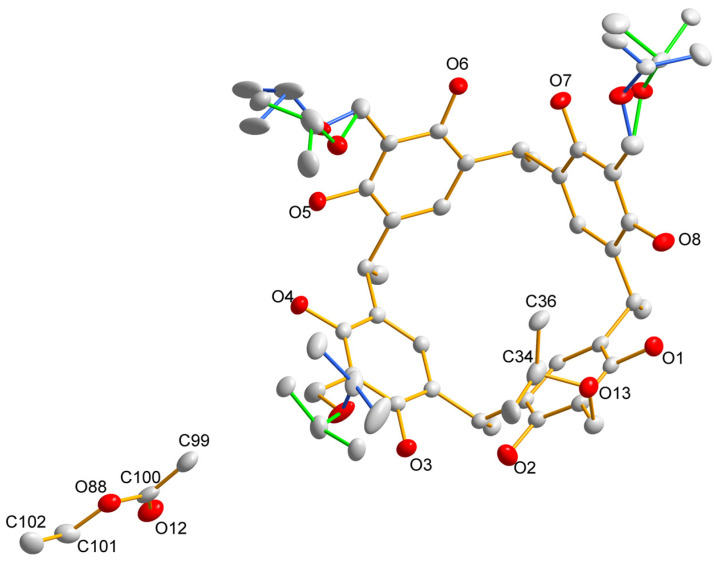
Asymmetric unit of compound **2a** with the adopted atom-numbering scheme. Hydrogen atoms and detailed atom labels are omitted for clarity. All non-hydrogen atoms are shown as thermal ellipsoids at 30% probability. Parts A and B of the disordered isopropoxy groups are highlighted in green and blue, respectively.

**Figure 4:**
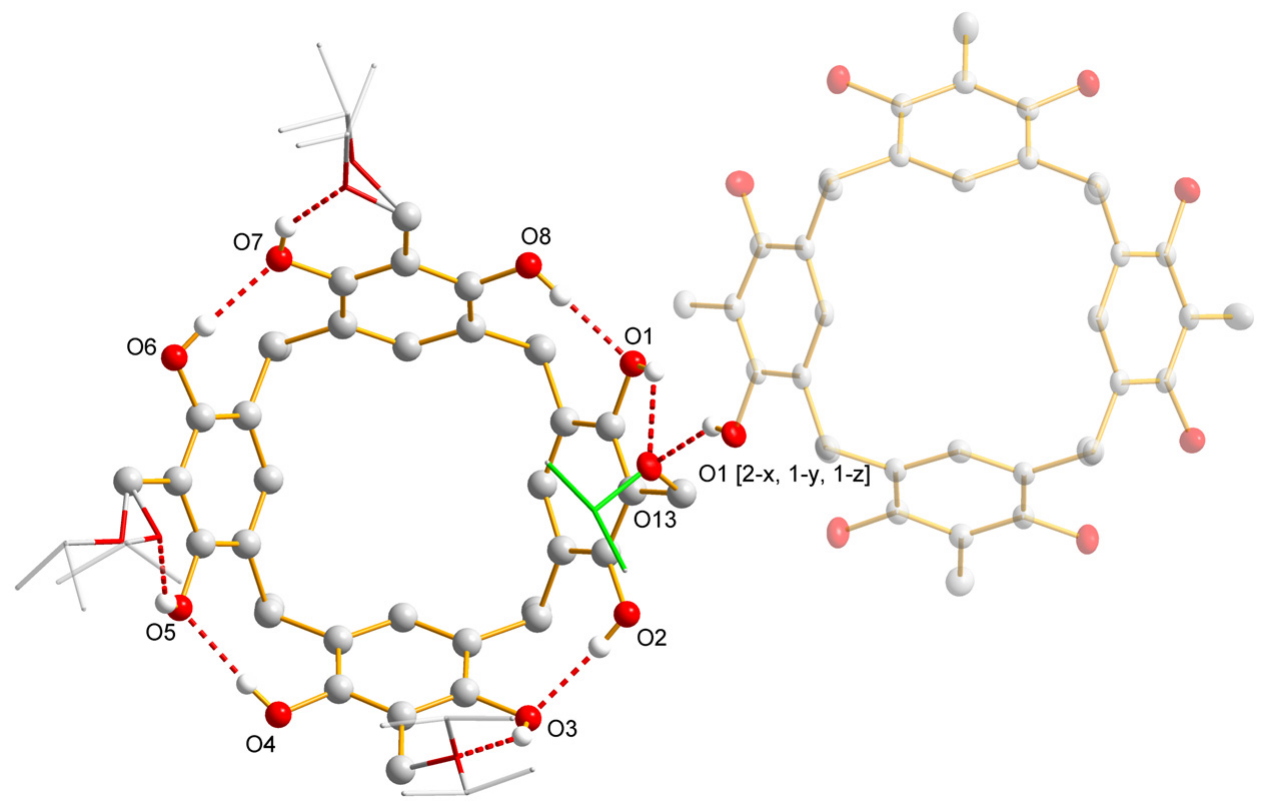
Hydrogen bonding interactions stabilizing the resorcinarene structure.

Crystallographic data and detailed information regarding the structure solution and refinement for compound **2a** are presented in Table 2. Selected bond parameters are summarized in Table 3. Crystals of compound **2a** belong to the triclinic crystal system and crystallize in the space group P−1. The asymmetric unit of **2a** comprises two molecules: one resorcinarene and one solvent molecule—ethyl acetate (Figure 3). The crystal structure shows a highly ordered resorcinarene framework stabilized by numerous intramolecular O–H⋯O hydrogen bonds (Figure 4). Three out of the four isopropoxy groups (except for the O13–C34–C35–C36 fragment) exhibit significant positional disorder.

This is likely due to the fact that these groups engage only in intramolecular hydrogen bonding (see Table 3), offering limited structural anchoring. In contrast, the isopropoxy group containing the oxygen atom O13 plays a key structural role as a hydrogen bond acceptor in two significant interactions: one intramolecular (O1–H100⋯O13) and one intermolecular, where the hydrogen donor is a symmetry-related molecule (O1[2–x, 1–y, 1–z]) (Figure 4).

**Figure 5:**
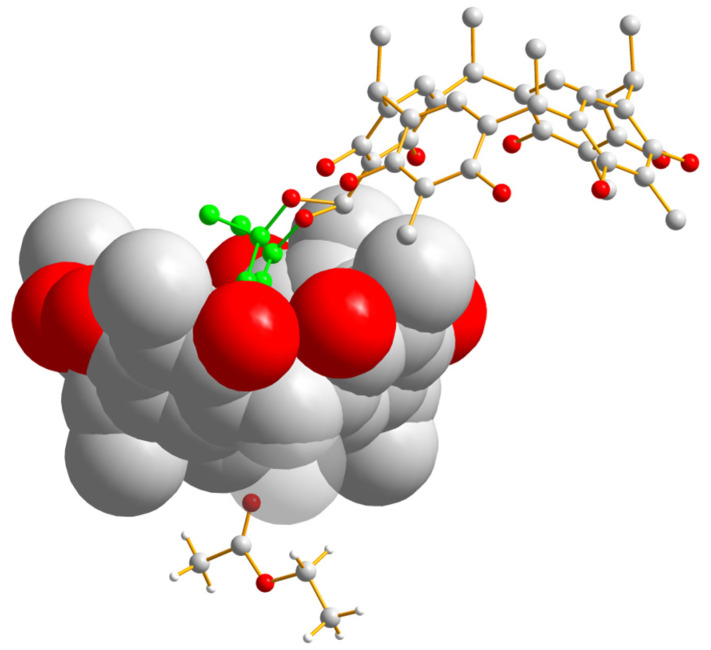
Penetration of a disordered isopropoxy group (green) from a neighboring molecule into the cavity of the resorcinarene structure.

This strong intermolecular interaction promotes direct contact between neighboring resorcinarene molecules. As a result of this interaction, the affected isopropoxy group adopts a conformation bent inward toward the resorcinarene cavity, with a characteristic torsion angle C5–C33–O13–O34 = 69.47°. Such a conformation is likely to hinder the access of guest molecules to the interior of the resorcinarene (Figure 5), while the remaining isopropoxy groups extend outward from the molecular framework.

These structural observations are particularly valuable from the perspective of crystal engineering, as they demonstrate the dual behavior of isopropoxy substituents within a single molecule. On one hand, isopropoxy groups engaged only in intramolecular interactions display greater conformational freedom and disorder; on the other, those involved in strong intermolecular hydrogen bonding contribute to crystal stability and likely drive nucleation and subsequent crystallization. Interestingly, despite the ethyl acetate molecule being located near the resorcinarene, it does not participate in any significant interactions, not even weak C–H⋯O contacts but rather occupies the interstitial spaces between bulky resorcinarene molecules (Figure 5). An analysis of the packing within the crystal of compound **2a** reveals a typical three-dimensional (3D) arrangement of molecules.

### 2.3. Biological Properties

The study evaluated the effects of selected compounds on four clinically significant bacterial species: *Staphylococcus aureus*, *Escherichia coli*, *Pseudomonas aeruginosa*, and *Bacillus subtilis*. *Staphylococcus aureus* is a Gram-positive, coccoid bacterium commonly found on human skin and mucous membranes [20]. It is responsible for a wide range of infections, including skin and wound infections, endocarditis, and nosocomial infections, particularly those associated with catheters and medical implants. The ability of *S. aureus* to form biofilms plays a critical role in the pathogenesis of device-related infections and significantly contributes to its resistance to antibiotic therapy. *Escherichia coli* is a Gram-negative rod-shaped bacterium that naturally inhabits the human gastrointestinal tract [21]. However, certain strains are pathogenic and can cause urinary tract infections, gastrointestinal infections, and sepsis. Biofilm formation by *E. coli* facilitates colonization of the urinary tract and hinders the elimination of uropathogenic strains. *Pseudomonas aeruginosa* is an opportunistic Gram-negative pathogen characterized by high levels of antibiotic resistance and a strong ability to form biofilms, particularly in the lungs of patients with cystic fibrosis, in burn wounds, and on the surfaces of medical devices [22]. *P. aeruginosa* biofilms exhibit a complex architecture and contain extracellular polymeric substances (EPS), which provide effective protection against antimicrobial agents. *Bacillus subtilis* is a Gram-positive soil-dwelling bacterium that is rarely pathogenic but can act as an opportunistic agent under certain conditions [23]. Due to its well-characterized regulatory mechanisms of biofilm formation, *B. subtilis* is widely used as a model organism in biofilm research.

#### 2.3.1. Biofilm and Its Role in Pathogenesis

A biofilm is an organized, three-dimensional structure in which microbial cells are embedded within an extracellular polymeric substance (EPS) matrix composed primarily of polysaccharides, proteins, and DNA. Biofilm formation is closely associated with the quorum sensing (QS) system, which regulates gene expression in response to cell population density. The ability of microorganisms to form biofilms is a key factor contributing to their pathogenicity, as it provides protection against environmental stressors and allows them to evade the host immune response. Moreover, the biofilm matrix limits the penetration of antimicrobial agents, promotes the formation of persister cells, and significantly complicates the treatment of infections, particularly chronic and hospital-acquired ones [24].

#### 2.3.2. Mechanism of Action and Antimicrobial Properties of Secondary and Tertiary Alcohols

Secondary and tertiary alcohols exhibit bactericidal activity through protein denaturation, dissolution of membrane lipids, and disruption of metabolic processes. These actions lead to membrane destabilization, increased permeability, and the loss of essential intracellular components, ultimately resulting in cell death. Secondary alcohols are highly effective against Gram-positive and Gram-negative bacteria, as well as certain viruses. In contrast, tertiary alcohols are generally less effective due to their more hydrophobic nature, which limits their ability to interact with microbial membranes. A key factor influencing the efficacy of alcohols is their concentration. Optimal antimicrobial activity is observed within the 60–90% range, where the presence of water facilitates efficient protein denaturation. Other important factors include contact time and the presence of organic matter (e.g., proteins or lipids), which can reduce alcohol effectiveness. Due to these properties, secondary alcohols are widely used in disinfection and are generally more effective than tertiary alcohols. Microorganisms, including bacteria and yeasts, have developed various protective mechanisms against the effects of alcohols. These include modifications of the cell membrane and wall structure, changes in transport and motility, activation of efflux pumps, synthesis of stress proteins, accumulation of protective compounds, and the formation of capsules and biofilms [25,26].

#### 2.3.3. Experimental Results and Analysis

The experimental results reveal a complex and varied impact of the tested alcohol derivatives on both the growth and biofilm formation of the analyzed bacterial strains. The evaluation was based on three parameters: cell growth (OD_600_), the amount of biofilm formed (CV_595_), and the CV_595_/OD_600_ ratio, which serves as an indicator of biofilm formation efficiency relative to cellular biomass (Table 4).

For *Staphylococcus aureus*, the highest CV_595_/OD_600_ ratio was observed for the 2-pentanol derivative **2c** (0.492), suggesting its significant role in stimulating biofilm formation. At the same time, this derivative had a moderate effect on bacterial growth, indicating an induction of stress response without a strong toxic effect. In contrast, the cyclohexanol derivative **2e** and the control sample (DMSO) exhibited the lowest ratio values, which may indicate a neutral or inhibitory effect on adhesion and biofilm formation processes. In the case of *Escherichia coli*, the most notable results were obtained for tertiary alcohol derivatives *t*-amyl **2g** (0.600) and *t*-butyl **2f** (0.513). Despite markedly inhibiting cell growth, these compounds significantly increased biofilm formation levels. This may reflect activation of survival mechanisms, such as enhanced production of the extracellular polymeric substance (EPS) matrix. Again, derivative **2e** and DMSO showed the lowest ratio values, confirming their relatively neutral impact on the bacteria. The results for *Pseudomonas aeruginosa* were particularly interesting. Regardless of the compound used, very low OD_600_ values were recorded, indicating strong growth inhibition. Simultaneously, biofilm formation levels were high, especially for the 2-pentanol derivative **2c** (CV_595_/OD_600_ = 10.54) and cyclohexanol derivative **2e** (8.92). Interestingly, the DMSO control also exhibited a very high ratio (20.89), suggesting this strain’s exceptional capacity to induce biofilm formation under stress conditions.

For *Bacillus subtilis*, the highest CV_595_/OD_600_ ratio was observed with derivative **2c** (0.715), indicating that this compound may act as a chemical signal inducing biofilm formation in Gram-positive bacteria. The lowest ratio was recorded in the control sample (DMSO), making it an appropriate reference point and suggesting minimal influence on biofilm formation in this strain. It is important to discuss the effect of the solvent used—5% dimethyl sulfoxide (DMSO). DMSO is a commonly employed solvent in biological studies; however, its impact on bacteria, especially at higher concentrations, can be ambiguous. In the analyzed samples, DMSO exhibited variable effects: for *S. aureus*, *E. coli*, and *B. subtilis*, the biofilm ratios were the lowest among all samples, suggesting a potentially inhibitory effect on biofilm formation. This may be due to the solvent’s protein-denaturing properties and disruption of membrane structures at elevated concentrations. Conversely, in *P. aeruginosa*, an exceptionally high CV_595_/OD_600_ value was observed, implying that in this case, DMSO might have acted as an environmental stressor, inducing biofilm formation as an adaptive response. It is also possible that DMSO influenced the binding capacity of crystal violet to the EPS matrix of this bacterium, leading to an overestimation of the result. A common conclusion across all strains is that the 2-pentanol derivative **2c** is a strong promoter of biofilm formation with relatively limited impact on bacterial growth. This effect may be related to activation of stress pathways, membrane disruption, or interference with the quorum sensing system. The cyclohexanol derivative **2e** and DMSO consistently produced the lowest biofilm ratios, suggesting their potentially neutral or anti-biofilm properties. Tertiary alcohol derivatives showed more variable effects depending on the strain but were generally also associated with increased biofilm formation.

#### 2.3.4. Hypothetical Molecular Mechanisms

Understanding the mechanisms by which aliphatic and cyclic alcohol derivatives affect bacterial cells and biofilm formation is crucial for interpreting the results and assessing their potential applications in antimicrobial strategies. Scientific literature suggests that these alcohols may influence bacteria through a range of complex molecular mechanisms, which vary depending on the chemical structure of the compound and the type of bacterial species involved [27,28]. One of the most important mechanisms is the direct interaction of alcohols with the cytoplasmic membrane. Alcohols can disrupt the structure and fluidity of the cell membrane, increasing its permeability and leading to a loss of cellular integrity. This effect is more pronounced with short, branched-chain alcohols, such as *t*-butyl and *t*-amyl alcohol, which more readily penetrate the lipid layers of the membrane and destabilize them. In response to membrane damage, bacteria often initiate a stress response, including increased production of biofilm matrix components, which may explain the observed increase in the CV_595_/OD_600_ ratio in *E. coli* and *S. aureus* [28,29,30].

Another key mechanism is the effect of alcohols on the quorum sensing (QS) system, which regulates intercellular communication and coordinates the expression of genes associated with biofilm formation. Some alcohol derivatives may act as mimics or inhibitors of signaling molecules. For example, structural similarities between 2-pentanols and natural QS ligands may lead to the activation of transcription of biofilm-related genes. Studies have shown that long aliphatic chains can interact with QS receptors in Gram-negative bacteria, such as *Pseudomonas aeruginosa*, resulting in enhanced EPS synthesis and increased biofilm resistance [31,32,33].

Alcohols can also influence the expression of genes involved in secondary metabolism, including the production of surfactants, enzymes, and proteases that facilitate cell adhesion to surfaces and intercellular interactions. In *Bacillus subtilis*, these compounds may activate regulatory pathways such as sinI/sinR, which control the switch between planktonic and biofilm modes of growth. The 2-pentanol derivative may act as an environmental signal inducing the transition to the biofilm state, which explains its strong effect observed across all analyzed strains. In the case of *Pseudomonas aeruginosa*, attention should be given to the possible interaction of alcohols with the GacS/GacA system and rsmY/rsmZ regulatory RNAs, which modulate the stability of mRNAs encoding biofilm-related factors. Furthermore, the increased biofilm formation accompanied by minimal growth suggests that these alcohol derivatives may act as inducers of persister cells—metabolically dormant forms capable of biofilm formation and survival under adverse conditions. It cannot be excluded that some alcohols also affect the composition and physicochemical properties of the EPS matrix, altering its affinity for the crystal violet dye. This phenomenon could partially explain the exceptionally high CV_595_/OD_600_ values observed for *P. aeruginosa* and DMSO [32,34,35].

In summary, the action of newly synthesized alcohol derivatives on biofilms may involve (i) destabilization of cell membranes, (ii) modulation of quorum sensing (QS) signaling systems, (iii) regulation of EPS gene expression and oxidative stress responses, and (iv) alterations in the biofilm matrix structure. Differences between Gram-positive and Gram-negative bacteria may arise from variations in cell wall architecture and specific regulatory systems. In the clinical context, understanding these mechanisms paves the way for targeted modulation of biofilms, aiming both at their inhibition and eradication in chronic infections.

## 3. Discussion

The presented study demonstrates a successful synthesis of novel alkoxylated resorcinarene derivatives bearing branched secondary and tertiary alcohol substituents. The structural diversity of the substituents significantly influenced not only the molecular architecture but also the biological properties of the compounds. While previous work on resorcinarenes focused largely on their synthetic versatility and supramolecular properties, this study extends their relevance to the biological realm by examining their effects on bacterial growth and biofilm formation.

The biological experiments revealed that the effect of the tested compounds on microbial behavior is multifaceted and highly strain-dependent. The data were interpreted based on three complementary parameters: optical density at 600 nm (OD_600_) to assess bacterial growth, crystal violet staining (CV_595_) to quantify biofilm biomass, and the CV/OD ratio as a normalized indicator of biofilm production relative to cell density.

Among all derivatives, compound **2c** (2-pentanol derivative) showed the most consistent biofilm-promoting effect, yielding the highest CV/OD ratios in *S. aureus*, *E. coli*, and *B. subtilis*. This indicates that **2c** may act not only as a metabolic stressor but possibly as a biofilm-inducing signal, potentially interfering with quorum sensing or membrane integrity. Interestingly, its influence on bacterial growth was modest, which suggests that the stimulation of biofilm production was not simply a byproduct of toxicity, but rather a specific biological response.

In contrast, compound **2e** (cyclohexanol derivative) and the DMSO control consistently exhibited low CV/OD values in three out of four strains, suggesting a neutral or potentially anti-biofilm effect. This behavior may be related to limited solubility or reduced interaction with bacterial membranes due to the compound’s more rigid and hydrophobic structure.

The most striking results were observed for *Pseudomonas aeruginosa*. Regardless of the compound applied, OD_600_ values remained extremely low, indicating strong inhibition of planktonic growth. However, CV_595_ absorbance remained high, especially for compound **2c** (CV/OD = 10.53) and the DMSO control (CV/OD = 20.89). This paradoxical pattern suggests that *P. aeruginosa* may respond to stress conditions not only by reducing growth but by activating a highly robust biofilm phenotype. The unusually high value for the DMSO control may reflect either a biological response to solvent-induced stress or an experimental artifact, possibly involving altered crystal violet binding to EPS. Follow-up controls with varying DMSO concentrations may help clarify this effect.

In *E. coli*, tertiary alcohol derivatives such as **2f** (*t*-butanol) and **2g** (*t*-amyl alcohol) induced both strong growth inhibition and elevated biofilm formation, supporting the hypothesis that tertiary alcohols act as environmental stressors that trigger survival mechanisms such as matrix overproduction. However, given the extremely low OD_600_ values, care must be taken in interpreting CV/OD ratios alone, as division by near-zero values can inflate results.

In *B. subtilis*, compound **2c** again stood out as the most potent inducer of biofilm (CV/OD = 0.715), suggesting that this compound may broadly affect Gram-positive species. The structurally well-characterized regulatory networks of *B. subtilis* could make it an ideal model for future mechanistic studies of biofilm induction by small molecules.

From a broader perspective, the data support the view that alkoxy-substituted resorcinarenes can modulate microbial behavior not only through direct toxicity but also by affecting regulatory pathways such as stress responses and quorum sensing. The structural features of the alkyl chains, particularly branching and hydrophobicity, appear to play a key role in determining biological outcomes.

Importantly, some derivatives (e.g., **2c** and **2f**) promoted biofilm formation without strong bactericidal effects. While such properties may initially seem undesirable from an antimicrobial standpoint, they could be strategically leveraged in materials science, for example, in designing surfaces that promote biofilm growth in beneficial contexts (e.g., bioreactors, probiotics). Conversely, compounds like **2e** could serve as starting points for anti-biofilm agents, given their neutral or suppressive profile.

In conclusion, the newly obtained biological data complement the structural findings and significantly expand the functional relevance of resorcinarene derivatives. The ability of selected compounds to selectively promote or suppress biofilm formation in clinically relevant bacteria highlights their potential as molecular tools or prototypes for antimicrobial strategies. Future work should aim to unravel the molecular mechanisms underlying these effects, potentially involving transcriptomic profiling, membrane interaction studies, and assessment of quorum sensing activity.

From a broader supramolecular chemistry perspective, our findings are consistent with previous studies reporting bioactivity in other macrocyclic systems such as calixarenes, cyclodextrins, and pillararenes. These compounds have been shown to modulate bacterial adhesion, membrane permeability, or biofilm formation through cavity-based interactions, amphiphilic surface presentation, or inclusion complexation mechanisms [12,13]. While the alkoxylated resorcinarenes presented here differ structurally and mechanistically from classical adsorbents, they contribute to this emerging class of biologically relevant host molecules. Compared to previously studied macrocycles, our derivatives offer structural tunability through alkyl chain variation, which may support future optimization for enhanced bioactivity or formulation compatibility.

## 4. Materials and Methods

^1^H and ^13^C NMR spectra were recorded using Bruker DRX 500 and Bruker Avance II 600 spectrometers (Bruker BioSpin GmbH, Rheinstetten, Germany), with CDCl_3_ and DMSO-d_6_ as solvents. Electrospray ionization mass spectra (ESI-MS) were obtained on a micrOTOF-Q II mass spectrometer (Bruker Daltonics, Billerica, MA, USA), and MALDI-TOF mass spectra were recorded on an Axima Performance instrument with a nitrogen laser (337 nm) (Shimadzu Corp., Kyoto, Japan) using 2-[(2E)-3-(4-tertbutylphenyl)-2-methylprop-2-enylidene]malononitrile (DCTB) as the matrix.

Diffraction intensity data for the single crystal of the new compound **2a** were collected at 115 K on a Rigaku XtaLAB Synergy-S diffractometer (Rigaku Corp., Tokyo, Japan) with mirror-monochromated Mo Kα radiation (λ = 0.71073 Å). Cell refinement and data reduction were performed using CrysAlisPro software (v40) (Rigaku Oxford Diffraction, Yarnton, UK) [36]. The positions of all non-hydrogen atoms were determined by direct methods using SHELXT version 2014/4 software (University of Göttingen, Göttingen, Germany). All non-hydrogen atoms were refined anisotropically using weighted full-matrix least-squares on F^2^. Refinement and further calculations were carried out using SHELXL software (ver. 2019/2) (University of Göttingen, Göttingen, Germany) [37,38]. All hydrogen atoms joined to carbon atoms were positioned with idealized geometries and refined using a riding model with Uiso(H) fixed at 1.2 Ueq (Carom). The figures were made using Diamond software, ver. 4.6.1 (Crystal Impact, Bonn, Germany) [39]. CCDC 2297228 contains the supplementary crystallographic data for **2a**.

Chromatographic separations were performed using silica gel 60 (Merck KGaA, Darmstadt, Germany; particle size 0.040–0.063 mm, 230–240 mesh). Melting points were determined on a Büchi B-540 melting point apparatus (Büchi Labortechnik AG, Flawil, Switzerland) and are uncorrected. All starting materials were purchased from Fluka (Honeywell, Seelze, Germany) and Merck KGaA (Darmstadt, Germany) and used without further purification. All solvents were of analytical grade and used as received.

### 4.1. General Procedure for the Synthesis

Resorcinarene (0.3 g, 0.55 mmol) was dissolved in acetonitrile (200 mL) as the solvent, followed by the addition of the appropriate secondary or tertiary alcohol (10 mL). Formaldehyde (0.25 mL, 3.3 mmol) and iminodiacetic acid (0.013 g, 0.097 mmol) were then added. The reaction mixture was stirred at 60 °C for approximately 72 h. After completion, the solvent was evaporated to dryness. The residue was dissolved in chloroform (100 mL) and washed with water (3 × 100 mL). The organic layer was dried and evaporated to afford the crude product as an oil containing residual alcohols. Following chromatographic purification, isolated yields of the products ranged from 62% to 74%.

#### 4.1.1. Tetra Iso-Propoxy Resorcinarene (**2a**)

The crude product was purified by column chromatography to yield a white solid product (0.23 g, 74% yield); R_f_ = 0.57 (ethyl acetate/hexane, 1:4); mp > 300 °C; ^1^H NMR (500 MHz, DMSO-d_6_) δ (ppm): 8.46 (s, 8H, ArOH), 7.32 (s, 4H, ArH), 4.52 (s, 8H, ArCH2O), 4.46 (q, *J* = 7.2 Hz, 4H, ArC*H*(CH_3_)Ar), 3.63 (m, 4H, OC*H*(CH_3_)_2_), 1.61 (d, *J* = 7.2 Hz, 12H, ArCH(C*H_3_*)Ar), 1.10 (d, *J* = 6.1 Hz, 24H, OCH(C*H_3_*)_2_); ^13^C NMR (126 MHz, DMSO-d_6_) δ (ppm): 150.04, 125.63, 124.31, 111.82, 71.26, 62.06, 28.78, 22.32, 21.01; HR-MS (ESI): *m*/*z* calculated for C_48_H_68_O_12_N [M + NH_4_]^+^ 850.4741268, found: 850.470552.

#### 4.1.2. Tetra *Sec*-Butoxy Resorcinarene (**2b**)

The crude product was purified by column chromatography to yield a white solid product (0.24 g, 73% yield); R_f_ = 0.69 (ethyl acetate/hexane, 1:4); mp > 300 °C; ^1^H NMR (500 MHz, CDCl_3_) δ (ppm): 9.02 (s, 8H, ArOH), 7.32 (s, 4H, ArH), 4.87 (d, 4H, *J* = 13.3 Hz, ArCH_2_O), 4.80 (d, *J* = 13.3 Hz, 4H, ArCH_2_O), 4.58 (q, *J* = 7.3 Hz, 4H, ArC*H*(CH_3_)Ar), 3.60–3.49 (m, 4H, OC*H*(CH_3_)CH_2_), 1.76 (d, *J* = 7.3 Hz, 12H, OCH(C*H_3_*)CH_2_), 1.69–1.49 (m, 8H, OCH(CH_3_)C*H_2_*), 1.21 (d, *J* = 6.75 Hz, 12H, ArCH(C*H_3_*)Ar), 0.92 (t, *J* = 7.50 Hz, 12H, OCH(CH_3_)CH_2_C*H_3_*); ^13^C NMR (126 MHz, CDCl_3_) δ (ppm): 149.42, 125.09, 121.97, 109.60, 77.71, 65.84, 28.73, 27.35, 19.86, 18.65, 9.40; HR-MS (ESI): *m*/*z* calculated for C_52_H_72_O_12_K [M + K]^+^ 927.4660592, found: 927.464951.

#### 4.1.3. Tetra *Sec*-Pentoxy Resorcinarene (**2c**)

The crude product was purified by column chromatography to yield a white solid product (0.23 g, 67% yield); R_f_ = 0.77 (ethyl acetate/hexane, 1:4); mp > 300 °C; ^1^H NMR (500 MHz, CDCl_3_) δ (ppm): 9.00 (s, 8H, ArOH), 7.30 (s, 4H, ArH), 4.86 (d, *J* = 13 Hz, 4H, ArCH_2_O), 4.78 (d, *J* = 13 Hz, 4H, ArCH_2_O), 4.56 (t, *J* = 7 Hz, 4H, ArC*H*(CH_3_)Ar), 3.58 (sextet, *J* = 6 Hz, 4H, OC*H*(CH_3_)CH_2_), 1.74 (d, *J* = 7 Hz, 12H, OCH(C*H_3_*)CH_2_CH_2_CH_3_), 1.63–1.57 (m, 4H, OCH(CH_3_)C*H_2_*CH_2_CH_3_), 1.48–1.41 (m, 4H, OCH(CH_3_)C*H_2_*CH_2_CH_3_), 1.40–1.29 (m, 8H, OCH(CH_3_)CH_2_C*H_2_*CH_3_), 1.20 (d, *J* = 6 Hz, 12H, ArCH(C*H_3_*)Ar), 0.92 (t, *J* = 7 Hz, 12H, OCH(CH_3_)CH_2_CH_2_C*H_3_*), ^13^C NMR (126 MHz, CDCl_3_); δ (ppm): 149.37, 125.06, 121.94, 109.58, 76.47, 65.88, 38.34, 27.33, 19.84, 19.13, 18.45, 14.11; HR-MS (ESI): *m*/*z* calculated for C_56_H_80_O_12_Na [M + Na]^+^ 967.554718, found: 967.551636.

#### 4.1.4. Tetra *Sec*-Hexoxy Resorcinarene (**2d**)

The crude product was purified by column chromatography to yield a white solid product (0.23 g, 63% yield); R_f_ = 0.80, (ethyl acetate/hexane, 1:4); mp > 300 °C; ^1^H NMR (600 MHz, DMSO-d_6_) δ (ppm): 8.52 (s, 8H, ArOH), 7.37 (s, 4H, ArH), 4.61 (d, *J* = 11.3 Hz, 4H, ArCH_2_O), 4.51 (d, *J* = 11.4 Hz, 4H, ArCH_2_O), 4.48 (q, *J* = 6.9 Hz, 4H, ArC*H*(CH_3_)Ar), 3.50 (m, 4H, CH_3_C*H*CH_2_(CH_2_)_2_CH_3_), 1.65 (d, *J* = 7.1 Hz, 12H, ArCH(C*H_3_*)Ar), 1.54–1.45 (m, 4H, CH_3_CHC*H_2_*(CH_2_)_2_CH_3_), 1.41–1.33 (m, 4H, CH_3_CHC*H_2_*(CH2)_2_CH_3_), 1.32–1.21 (m, 16H, CH_3_CHCH_2_(*CH_2_*)_2_CH_3_), 1.11 (d, *J* = 6.1 Hz, 12H, C*H_3_*CHCH_2_(CH_2_)_2_CH_3_), 0.87 (t, *J* = 6.4 Hz, 12H, CH_3_CHCH_2_(CH_2_)_2_C*H_3_*); ^13^C NMR (151 MHz, DMSO-d_6_) δ (ppm): 148.90, 124.51, 123.08, 110.61, 73.92, 61.20, 34.89, 28.31, 26.25, 21.53, 19.79, 18.62, 13.27; HR-MS (ESI): *m*/*z* calculated for C_60_H_88_O_12_K [M + K]^+^ 1039.5912528, found: 1039.592197.

#### 4.1.5. Tetra Cyclo-Hexoxy Resorcinarene (**2e**)

The crude product was purified by column chromatography to yield a white solid product (0.23 g, 62% yield); R_f_ = 0.85, (ethyl acetate/hexane, 1:4); mp > 300 °C; ^1^H NMR (500 MHz, DMSO-d_6_) δ (ppm): 8.47 (s, 8H, ArOH), 7.29 (s, 4H, ArH), 4.54 (s, 8H, ArCH_2_O), 4.44 (q, *J* = 7.1 Hz, 4H, ArC*H*(CH_3_)Ar), 3.38–3.33 (m, 4H, OC*H*(CH_2_)_2_(CH_2_)_2_CH_2_), 1.86–1.80 (m, 8H, OCH(C*H*_2_)_2_(CH_2_)_2_CH_2_), 1.59 (d, *J* = 7.2 Hz, 12H, ArCH(C*H_3_*)Ar), 1.47–1.41 (m, 8H, OCH(C*H_2_*)_2_(CH_2_)_2_CH_2_), 1.28–1.11 (m, 24H, OCH(CH_2_)_2_(C*H_2_*)_2_C*H_2_*); ^13^C NMR (126 MHz, DMSO-d_6_) δ (ppm): 150.07, 125.59, 124.25, 111.77, 76.89, 62.01, 31.90, 28.76, 25.76, 23.79, 20.97; HR-MS (ESI): *m*/*z* calculated for C_60_H_84_O_12_N [M + NH_4_]^+^ 1010.5993204, found 1010.590201.

#### 4.1.6. Tetra *Tert*-Butyloxy Resorcinarene (**2f**)

The crude product was purified by column chromatography to yield a white solid product (0.12 g, 36% yield); R_f_ = 0.53 (ethyl acetate/hexane, 1:4); mp > 300 °C; ^1^H NMR (500 MHz, CDCl_3_) δ (ppm): 9.16 (s, 8H, ArOH), 7.27 (s, 4H, ArH), 4.77 (s, 8H, ArCH_2_O), 4.54 (q, *J* = 7.4 Hz, 4H, ArC*H*(CH_3_)Ar), 1.72 (d, *J* = 7.8 Hz, 12H, ArCH(C*H_3_*)Ar), 1.29 (s, 36H, OC(CH_3_)_3_); ^13^C NMR (126 MHz, CDCl_3_) δ (ppm): 149.26, 125.06, 121.69, 110.02, 75.73, 60.45, 29.68, 27.29, 19.88; HR-MS (ESI): *m*/*z* calculated for C_52_H_72_O_12_Na [M + Na]^+^ 911.4921212, found: 911.492667.

#### 4.1.7. Tetra *Tert*-Amyloxy Resorcinarene (**2g**)

The crude product was purified by column chromatography to yield a white solid product (0.10 g, 29% yield); R_f_ = 0.85 (ethyl acetate/hexane, 1:4); mp > 300 °C; ^1^H NMR (500 MHz, CDCl_3_) δ (ppm): 9.23 (s, 8H, ArOH), 7.27 (s, 4H, ArH), 4.74 (s, 8H, ArCH_2_O), 4.54 (q, *J* = 7.4 Hz, 4H, ArC*H*(CH_3_)Ar), 1.73 (d, *J* = 7.8 Hz, 12H, ArCH(C*H_3_*)Ar), 1.60 (q, *J* = 7.9 Hz, 8H, O(CH_3_)_2_C*H_2_*CH_3_), 1.23 (s, 24H, O(C*H_3_*)_2_CH_2_CH_3_), 0.89 (q, *J* = 7.6 Hz, 12H, O(CH_3_)_2_CH_2_C*H_3_*); ^13^C NMR (126 MHz, CDCl_3_) δ (ppm): 149.28, 125.05, 121.64, 109.98, 77.92, 60.12, 32.70, 27.31, 24.66, 19.86, 8.33; HR-MS (ESI): *m*/*z* calculated for C_56_H_80_O_12_Na [M + Na]^+^ 967.554718, found: 967.554282.

### 4.2. Model Organisms and Experimental Design

*Escherichia coli*, *Pseudomonas aeruginosa* (Gram-negative), and *Staphylococcus aureus* and *Bacillus subtilis* (Gram-positive) were chosen as model organisms due to their structural differences and relevance in antimicrobial research. After reactivation from −80 °C glycerol stocks and triple passage on LB agar, single colonies were suspended in saline and adjusted to a 0.5 McFarland standard. A microdilution assay was performed with the tested compounds (0.1 mg/mL in DMSO) against all strains. DMSO served as a control. Cultures were incubated at 37 °C for 24 h, and OD_600_ was measured.

The amount of biofilm produced by bacteria was assessed based on the measurement of crystal violet bound to the biofilm matrix during staining. Bacteria were cultivated in 96-well plates under previously described conditions. After the cultivation, the 96-well plates were rinsed three times with sterile physiological saline to remove planktonic cells and non-adherent biofilm fragments. The plates were then left to dry at room temperature for 1 h. Next, 250 µL of 0.1% crystal violet solution was added to each well. Staining was carried out for 30 min at room temperature. After that, the crystal violet solution was removed, and the wells were rinsed three times with sterile physiological saline. The 96-well plates were left to air dry completely for 1 h. Subsequently, 250 µL of 33% acetic acid solution was added to each well to elute the crystal violet from the biofilm matrix. Absorbance was measured spectrophotometrically at a wavelength of 595 nm. The experiment was performed in three independent replicates. Measurements were conducted using the Infinity 200 Pro spectrophotometer (Tecan).

## 5. Conclusions

A novel Mannich-type methodology was successfully applied to introduce branched alkoxy groups into resorcinarene derivatives using secondary and tertiary alcohols. The resulting compounds were obtained in moderate to high yields and were thoroughly characterized by spectroscopic and crystallographic methods. X-ray diffraction analysis confirmed the role of intramolecular and intermolecular hydrogen bonding in stabilizing the macrocyclic structure and modulating conformational behavior. Moreover, under specific conditions, mass spectrometric analysis revealed the formation of oligomeric supramolecular species, which may serve as precursors for future functional materials or self-assembled systems.

In addition to the synthetic and structural studies, selected derivatives (**2b**–**2f**) were evaluated for their biological activity. The data revealed significant structure-dependent effects on bacterial growth and biofilm formation in four clinically relevant strains. Notably, compound **2c** (2-pentanol derivative) emerged as a potent promoter of biofilm formation across multiple species, with limited cytotoxicity. In contrast, compound **2e** (cyclohexanol derivative) and the DMSO control exhibited neutral or potentially anti-biofilm properties. These findings suggest that specific structural features of the alkoxy substituents, such as branching and hydrophobicity, can modulate microbial responses, potentially by interfering with membrane integrity or quorum sensing.

This study highlights the dual functional potential of alkoxylated resorcinarenes as both supramolecular building blocks and modulators of microbial behavior. The integration of synthetic design with biological evaluation opens new avenues for the development of resorcinarene-based systems in areas such as biofilm control, targeted delivery, or the creation of smart materials responsive to biological stimuli.

## Figures and Tables

**Table 1:** Yields of alkoxymethylene resorcinarene derivatives.

Compound	R	Yields
**2a**	iso-propyl	74%
**2b**	*sec*-butyl a	73%
**2c**	*sec*-pentyl	67%
**2d**	*sec*-hexyl	63%
**2e**	cyclohexyl	62%
**2f**	*tert*-butyl	36%
**2g**	*tert*-amyl	29%

**Table 2:** Crystal data and structure refinement for **2a**.

Empirical Formula	C_52_H_72_O_14_
Formula weight	921.09
Temperature	115(2) K
Wavelength	0.71073 Å
Crystal system	Triclinic
Space group	P−1
Unit cell dimension	a = 12.4393(4) Å α = 100.084(2)° b = 13.1667(4) Å β = 96.994(2)° c = 16.4740(4) Å γ = 108.623(3)°
Volume	2471.34(13) Å^3^
Z	2
Density (calculated)	1.238 Mg/m^3^
Absorption coefficient	0.089 mm^−1^
F(000)	992
Crystal size	0.150 × 0.120 × 0.100 mm^3^
Theta range for data collection	2.558 to 30.923°
Index ranges	−17 ≤ h ≤ 13, −18 ≤ k ≤ 18, −22 ≤ l ≤ 22
Reflections collected	37,979
Independent reflections	12,570 [R(int) = 0.0349]
Completeness to theta = 25.242°	99.7%
Refinement method	Full-matrix least-squares on F^2^
Data/restraints/parameters	12,570/5/713
Goodness-of-fit on F^2^	1.071
Final R indices [I > 2sigma(I)]	R1 = 0.0517, wR2 = 0.1272
R indices (all data)	R1 = 0.0749, wR2 = 0.1388
Largest diff. peak and hole	0.619 and −0.571 e/Å^3^

**Table 3:** Hydrogen bonds for **2a** [Å and °].

D-H⋯A	d(D-H)	d(H⋯A)	d(D⋯A)	<(DHA)
O(1)-H(1OO)⋯O(13)	0.84	2.43	3.0677(15)	133.3
O(1)-H(1OO)⋯O(13)#1	0.84	1.98	2.7107(14)	145.2
O(3)-H(3O)⋯O(10)	0.84	1.88	2.6194(17)	145.9
O(7)-H(7O)⋯O(12A^a)	0.84	1.89	2.612(3)	143.5
O(7)-H(7O)⋯O(12B^b)	0.84	1.77	2.517(3)	146.7
O(6)-H(6O)⋯O(7)	0.84	1.91	2.7430(16)	175.1
O(2)-H(2O)⋯O(3)	0.84	1.93	2.7496(16)	165.9
O(5)-H(5O)⋯O(11A^e)	0.84	1.88	2.597(3)	142.8
O(5)-H(5O)⋯O(11B^f)	0.84	1.82	2.534(5)	142.4
O(8)-H(8O)⋯O(1)	0.84	1.9	2.7406(15)	177.3
O(4)-H(4O)⋯O(5)	0.84	1.94	2.7721(15)	173.4

Symmetry transformations used to generate equivalent atoms: #1 −x + 2, −y + 1, −z + 1.

**Table 4:** Effect of resorcinarene derivatives (**2b**–**2f**) on bacterial growth and biofilm formation in selected strains after 24 h of incubation (OD_600_ and crystal violet staining).

Compounds	OD_600_ *	SD_OD600_ *	CV_595_ *	SD_CV595_ *	CV_595_/OD_600_ *
*S. aureus*					
**2b**	0.3869	0.0094	0.1227	0.0113	0.3172
**2c**	0.2817	0.0112	0.1386	0.0100	0.4921
**2e**	0.3259	0.0230	0.0956	0.0068	0.2935
**2f**	0.2666	0.0268	0.1100	0.0039	0.4126
**2g**	0.3010	0.0011	0.0991	0.0014	0.3293
DMSO 5%	0.3179	0.0087	0.0723	0.0022	0.2275
*E. coli*					
**2b**	0.3761	0.0087	0.1269	0.0037	0.3373
**2c**	0.2844	0.0197	0.1306	0.0023	0.4591
**2e**	0.3498	0.0219	0.0981	0.0044	0.2805
**2f**	0.2267	0.0171	0.1162	0.0027	0.5128
**2g**	0.1849	0.1372	0.1108	0.0054	0.5995
DMSO 5%	0.2693	0.0239	0.0728	0.0017	0.2702
*P. aeruginosa*					
**2b**	0.0470	0.0016	0.3741	0.0230	7.9589
**2c**	0.0428	0.0011	0.4513	0.0139	10.5354
**2e**	0.0256	0.0040	0.2281	0.0574	8.9218
**2f**	0.0220	0.0124	0.1759	0.0112	8.0076
**2g**	0.0236	0.0019	0.1312	0.0042	5.5672
DMSO 5%	0.0267	0.0035	0.5578	0.0090	20.8926
*B. subtilis*					
**2b**	0.2441	0.0192	0.1268	0.0041	0.5196
**2c**	0.1895	0.0089	0.1356	0.0036	0.7154
**2e**	0.2350	0.0119	0.1093	0.0076	0.4649
**2f**	0.1775	0.0214	0.1033	0.0043	0.5821
**2g**	0.2805	0.0178	0.1165	0.0027	0.4152
DMSO 5%	0.2612	0.0081	0.0788	0.0022	0.3016

* OD_600_—optical density of the culture after 24 h (indicator of cell growth), SD _OD600_—standard deviation for OD_600_, CV_595_—absorbance after crystal violet staining (indicator of biofilm formation), SD_CV595_—standard deviation for CV_595_, CV_595_/OD_600_—ratio of biofilm to cellular biomass.

## Data Availability

The data supporting the findings of this study are available in the Supplementary Materials associated with this article. Crystallographic data for compound **2a** have been deposited at the Cambridge Crystallographic Data Centre (CCDC) under deposition number 2297228 and can be obtained free of charge via www.ccdc.cam.ac.uk or by emailing data_request@ccdc.cam.ac.uk.

## Supplementary Materials

The following supporting information can be downloaded at: https://www.mdpi.com/article/10.3390/molecules30153304/s1.
