# Supplementary material for: Structure-Activity Relationships in Alkoxylated Resorcinarenes: Synthesis, Structural Features, and Bacterial Biofilm-Modulating Properties
Source: Molecules. 2025 Aug 7;30(15):3304. doi: 10.3390/molecules30153304 (PMC12348843; doi:10.3390/molecules30153304)
Supplement: Supplementary file 1 [file molecules-30-03304-s001.zip › molecules-3780208-supplementary.pdf]

Structure–Activity Relationships in Alkoxylated Resorcinarenes: Synthesis, Structural Features, and Bacterial Biofilm-Modulating Properties

Supporting Information

Contents

Mass spectra of products obtained from the Mannich reaction catalyzed by iminodiacetic acid.................................2
^{1}H, ^{13}C NMR and HR-MS spectra of compounds **2a**-**g**..5
Single-crystal X-ray diffraction data for compound **2a**...19

Mass spectra of products obtained from the Mannich reaction catalyzed by iminodiacetic acid

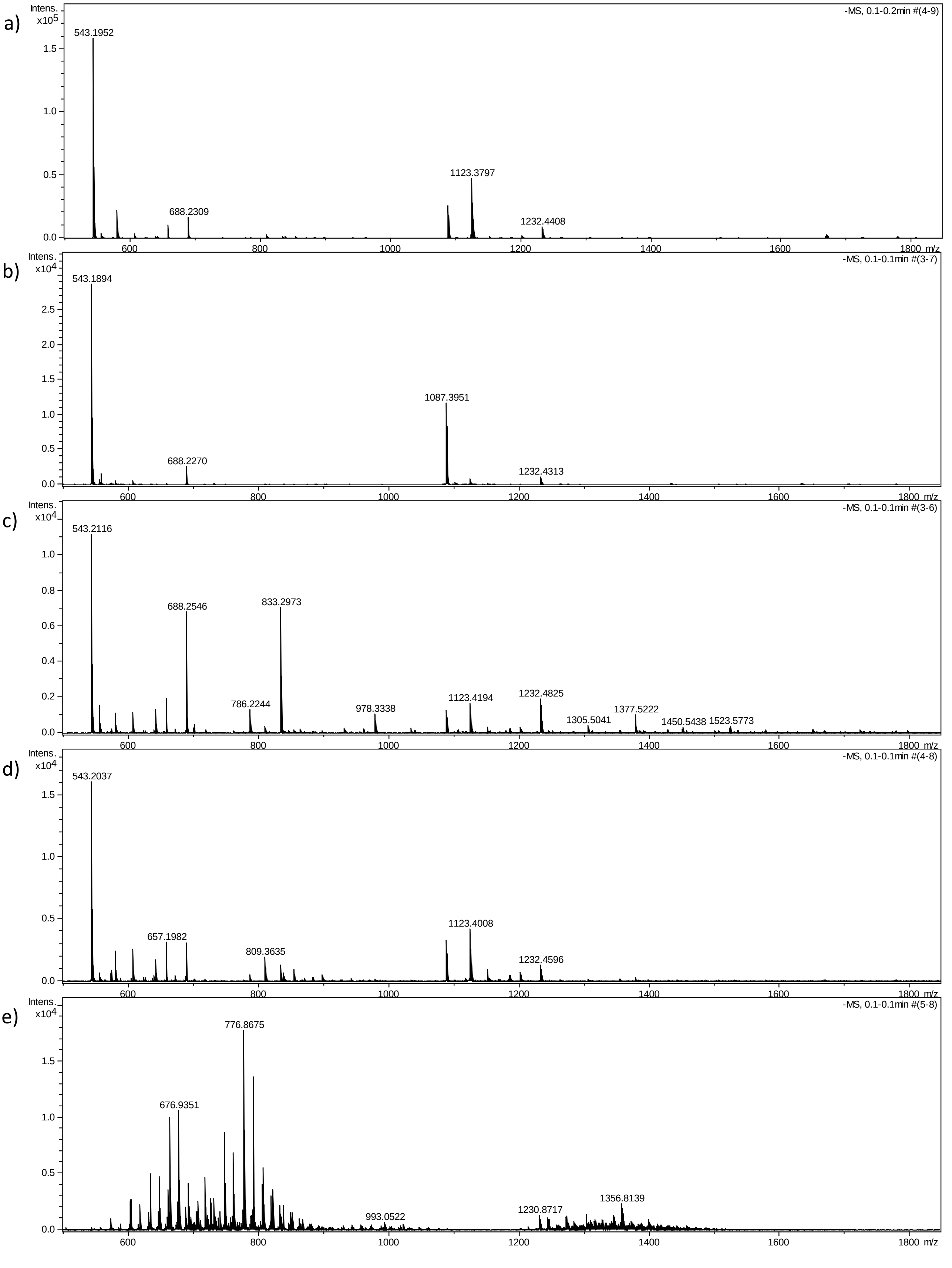

Figure S1. Negative-ion ESI-TOF mass spectrum of the reaction mixture recorded at a) 15 minutes, b) 30 minutes, c) 45 minutes d) 60 minutes and e) 90 minutes.

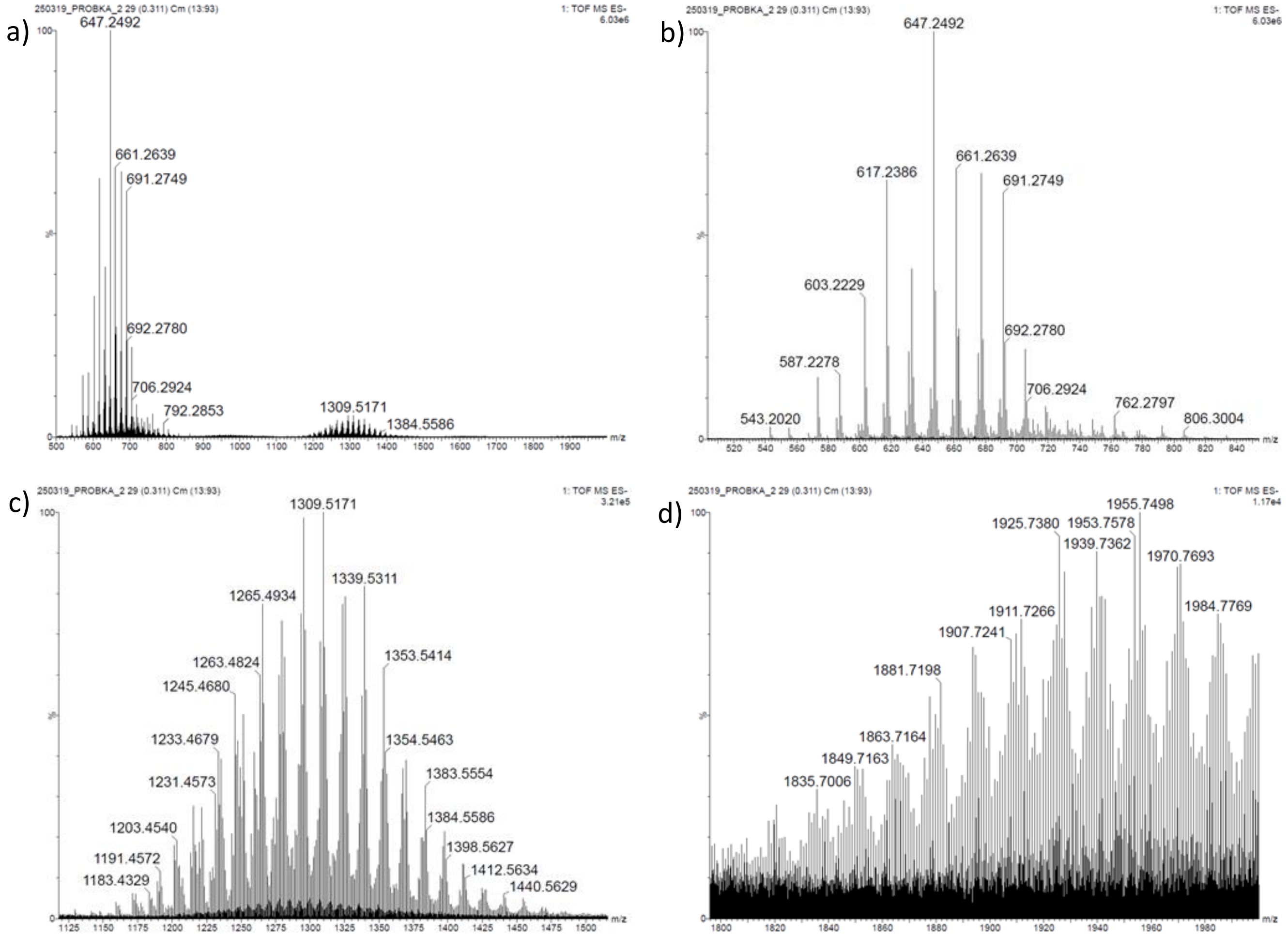

Figure S2. Negative-ion ESI-TOF mass spectra of oligomeric products from the Mannich reaction catalyzed by iminodiacetic acid (a), with enlarged regions shown in b–d.

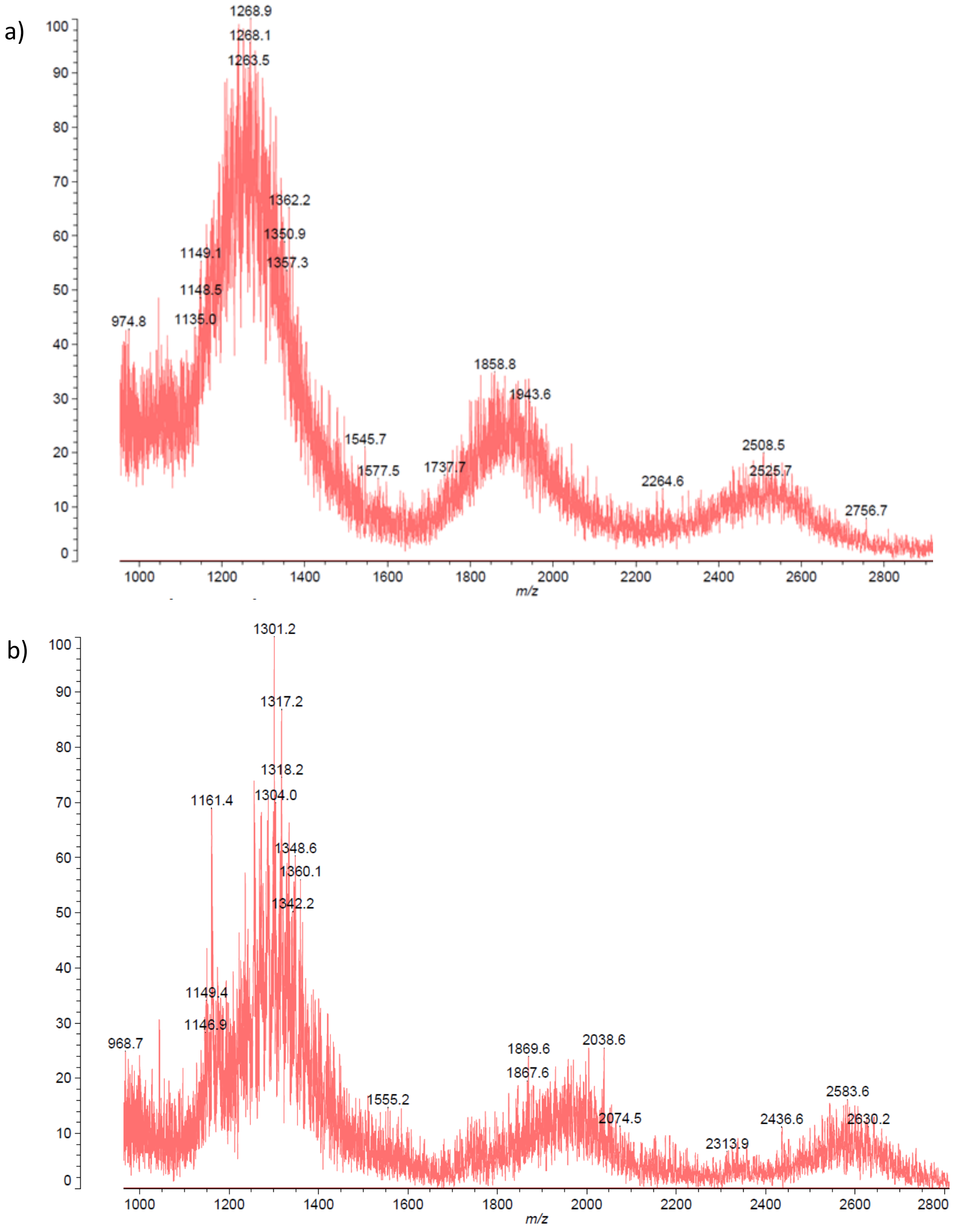

Figure S3. Positive-ion MALDI-TOF mass spectra of oligomeric products from the Mannich reaction catalyzed by iminodiacetic acid, using a) 2,5-dihydroxybenzoic acid (DHB) and b) DCTB as matrices.

^{1}H, ^{13}C NMR and HR-MS spectra of compounds 2a-g.

tetra iso-propoxy resorcinarene (2a). The crude product was purified by column chromatography to yield white solid product (0.23 g, 74% yield); R_f = 0.57 (ethyl acetate/hexane, 1:4); mp > 300 °C; ^{1}H NMR (500 MHz, DMSO-d_6) δ (ppm): 8.46 (s, 8H, ArOH), 7.32 (s, 4H, ArH), 4.52 (s, 8H, $ArCH_2O$), 4.46 (q, J = 7.2 Hz, 4H, ArC*H*(CH_3)Ar), 3.63 (m, 4H, OC*H*(CH_3)$_2$), 1.61 (d, J = 7.2 Hz, 12H, ArCH(C*H*$_3$)Ar), 1.10 (d, J = 6.1 Hz, 24H, OCH(C*H*$_3$)$_2$); ^{13}C NMR (126 MHz, DMSO-d_6) δ (ppm): 150.04, 125.63, 124.31, 111.82, 71.26, 62.06, 28.78, 22.32, 21.01; HR-MS (ESI): *m/z* calculated for $C_{48}H_{68}O_{12}N$ [M+NH$_4$]$^+$ 850.4741268, found: 850.470552.

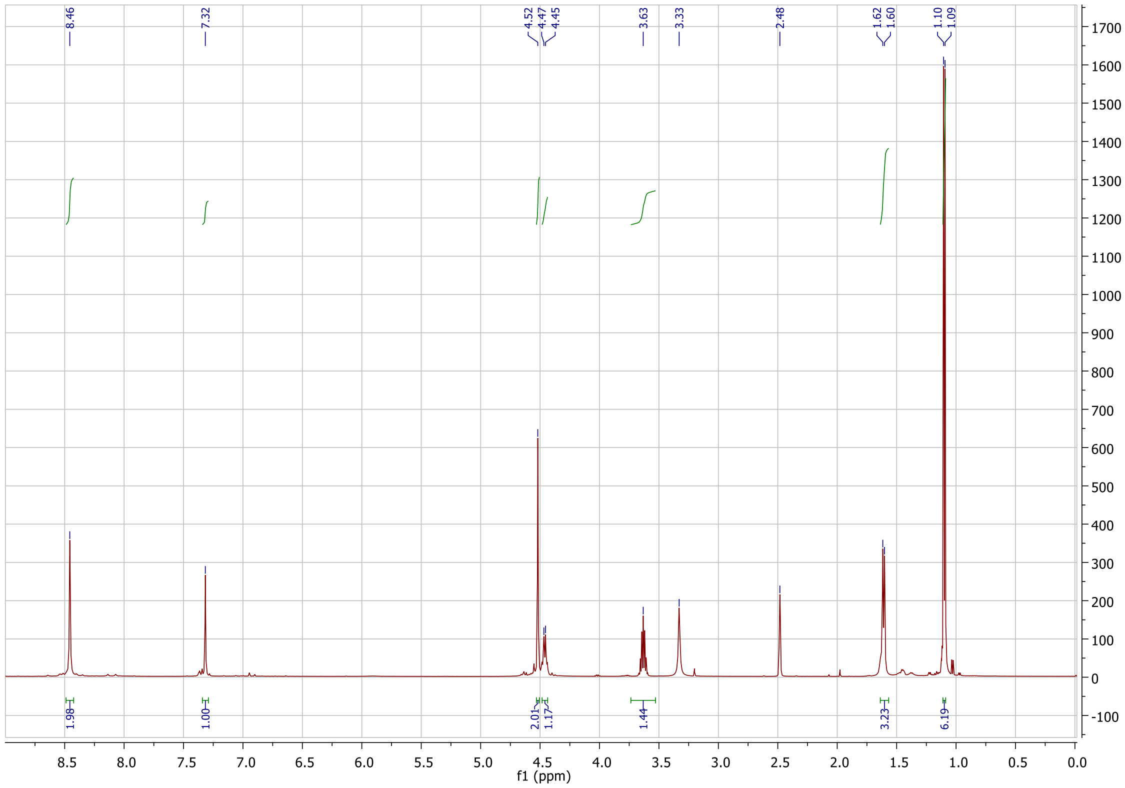

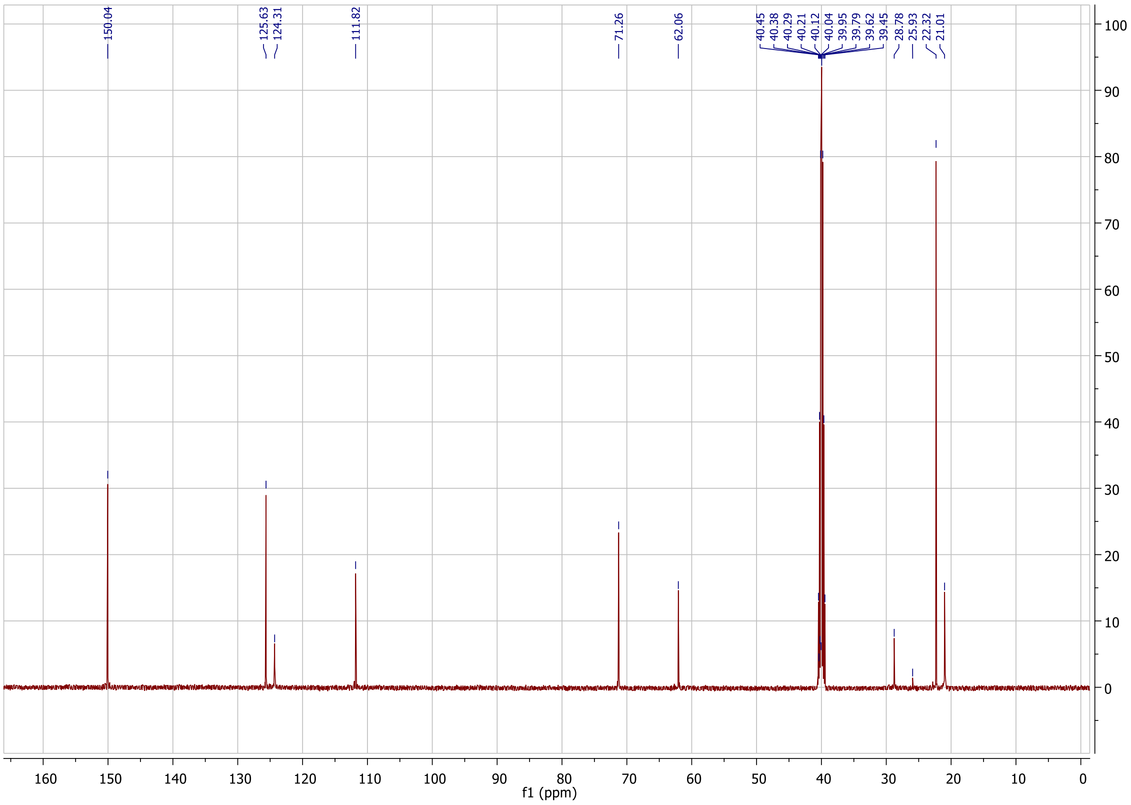

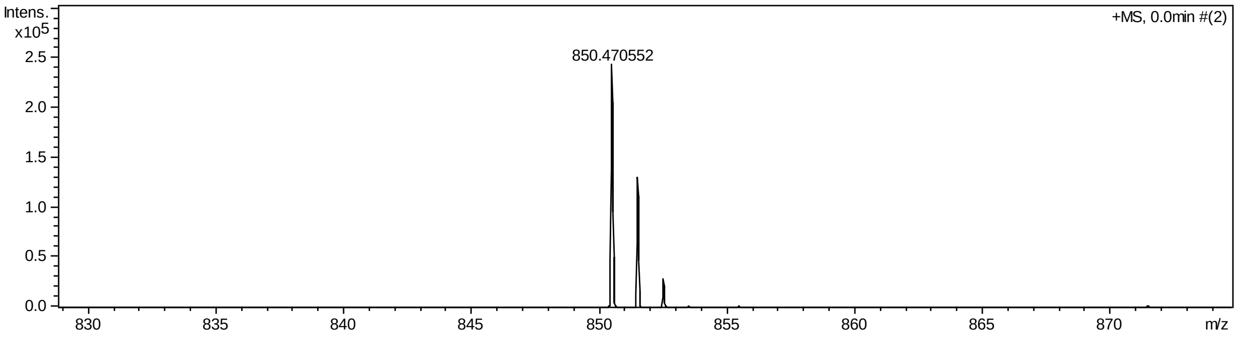
Intens.
x10^5
+MS, 0.0min #(2)
850.470552
m/z

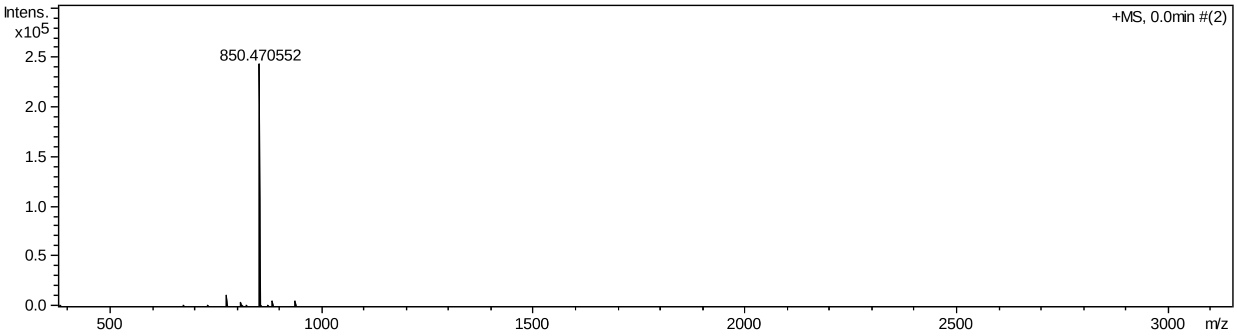
Intens.
x10^5
+MS, 0.0min #(2)
850.470552
m/z

tetra sec-butoxy resorcinarene (**2b**). The crude product was purified by column chromatography to yield white solid product (0.24 g, 73% yield); R_f = 0.69 (ethyl acetate/hexane, 1:4); mp > 300 °C; ^{1}H NMR (500 MHz, $CDCl_3$) δ (ppm): 9.02 (s, 8H, ArOH), 7.32 (s, 4H, ArH), 4.87 (d, 4H, *J* = 13.3 Hz, $ArCH_2O$), 4.80 (d, *J* = 13.3 Hz, 4H, $ArCH_2O$), 4.58 (q, *J* = 7.3 Hz, 4H, ArC*H*(CH_3)Ar), 3.60-3.49 (m, 4H, OC*H*(CH_3)CH_2), 1.76 (d, *J* = 7.3 Hz, 12H, OCH(CH_3)CH_2), 1.69-1.49 (m, 8H, OCH(CH_3)CH_2), 1.21 (d, *J* = 6.75 Hz, 12H, ArCH(CH_3)Ar), 0.92 (t, *J* = 7.50 Hz, 12H, OCH(CH_3)CH_2CH_3); ^{13}C NMR (126 MHz, $CDCl_3$) δ (ppm): 149.42, 125.09, 121.97, 109.60, 77.71, 65.84, 28.73, 27.35, 19.86, 18.65, 9.40; HR-MS (ESI): *m/z* calculated for $C_{52}H_{72}O_{12}K$ [M+K]$^+$ 927.4660592, found: 927.464951.

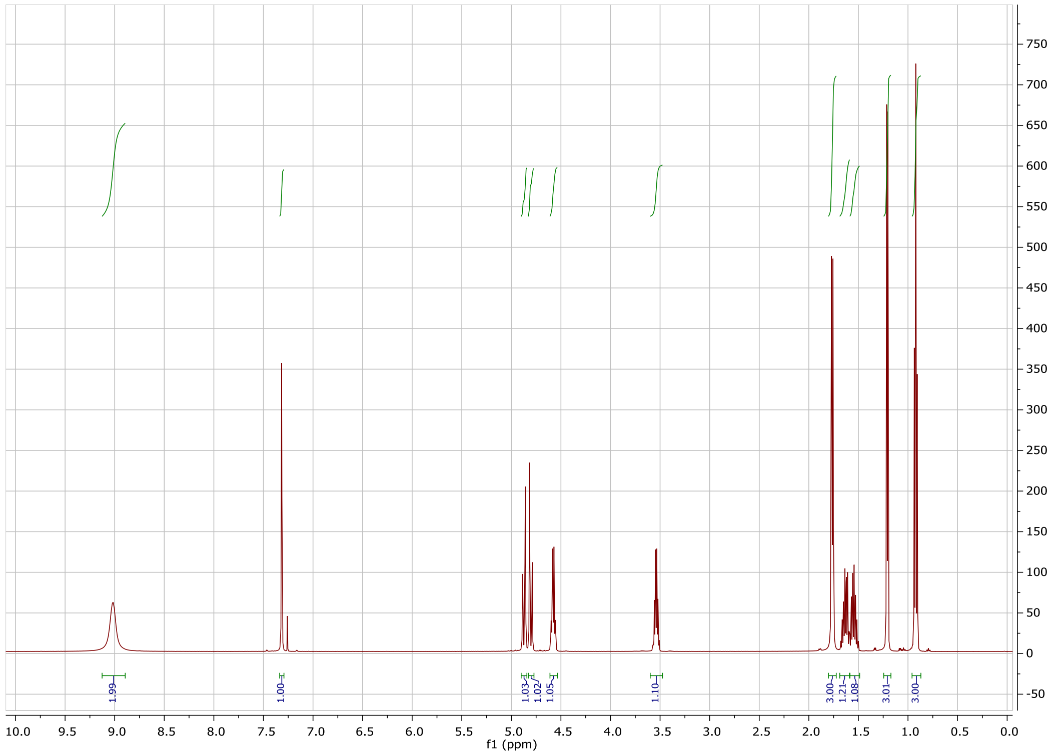

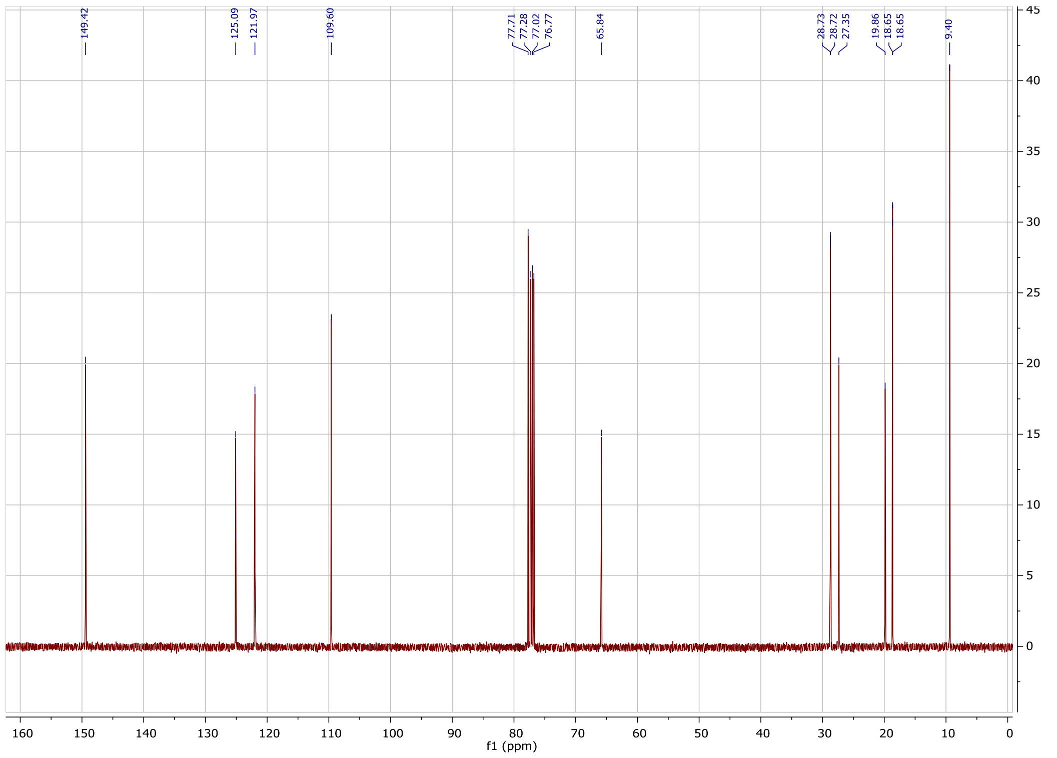

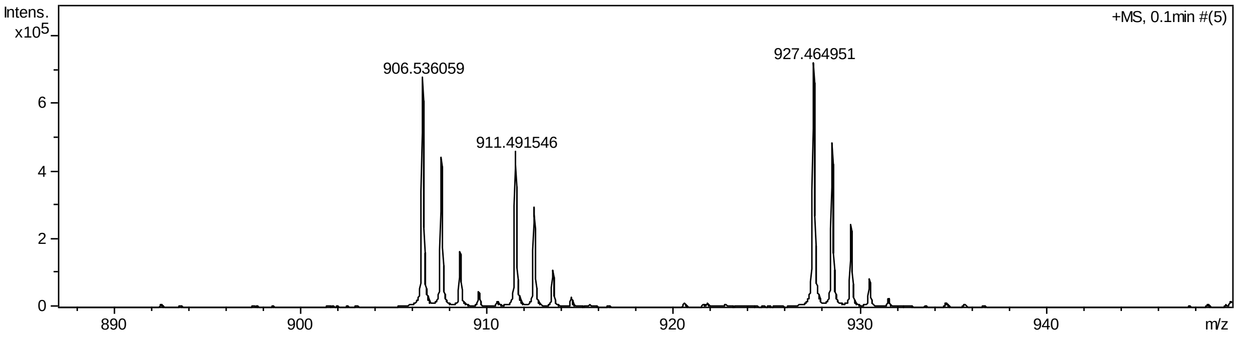
Intens.
x10^5
+MS, 0.1min #(5)
906.536059
911.491546
927.464951
m/z

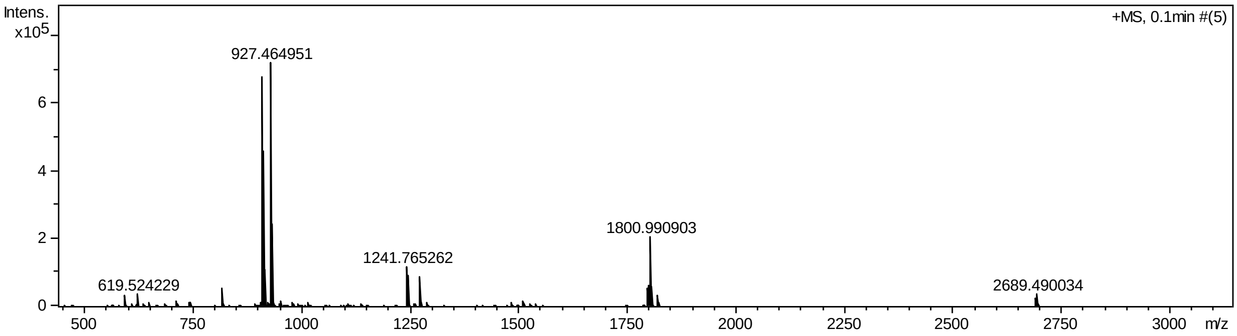
Intens.
x10^5
+MS, 0.1min #(5)
927.464951
619.524229
1241.765262
1800.990903
2689.490034
m/z

tetra sec-pentoxy resorcinarene (2c). The crude product was purified by column chromatography to yield white solid product (0.23g, 67% yield): R_f = 0.77 (ethyl acetate/hexane, 1:4); mp > 300 °C; ^{1}H NMR (500 MHz, $CDCl_3$) δ (ppm): 9.00 (s, 8H, ArOH), 7.30 (s, 4H, ArH), 4.86 (d, *J* = 13 Hz, 4H, $ArCH_2O$), 4.78 (d, *J* = 13 Hz, 4H, $ArCH_2O$), 4.56 (t, *J* = 7 Hz, 4H, ArC*H*(CH_3)Ar), 3.58 (sextet, *J* = 6 Hz, 4H, OC*H*(CH_3)CH_2), 1.74 (d, *J* = 7 Hz, 12H, OCH(CH_3)$CH_2CH_2CH_3$), 1.63-1.57 (m, 4H, OCH(CH_3)C$H_2$$CH_2CH_3$), 1.48-1.41 (m, 4H, OCH(CH_3)C$H_2$$CH_2CH_3$), 1.40-1.29 (m, 8H, OCH(CH_3)CH_2C$H_2$$CH_3$), 1.20 (d, *J* = 6 Hz, 12H, ArCH(CH_3)Ar), 0.92 (t, *J* = 7 Hz, 12H, OCH(CH_3)CH_2CH_2CH_3), ^{13}C NMR (126 MHz, $CDCl_3$); δ (ppm): 149.37, 125.06, 121.94, 109.58, 76.47, 65.88, 38.34, 27.33, 19.84, 19.13, 18.45, 14.11; HR-MS (ESI): *m/z* calculated for $C_{56}H_{80}O_{12}Na$ $[M+Na]^+$ 967.554718, found: 967.551636.

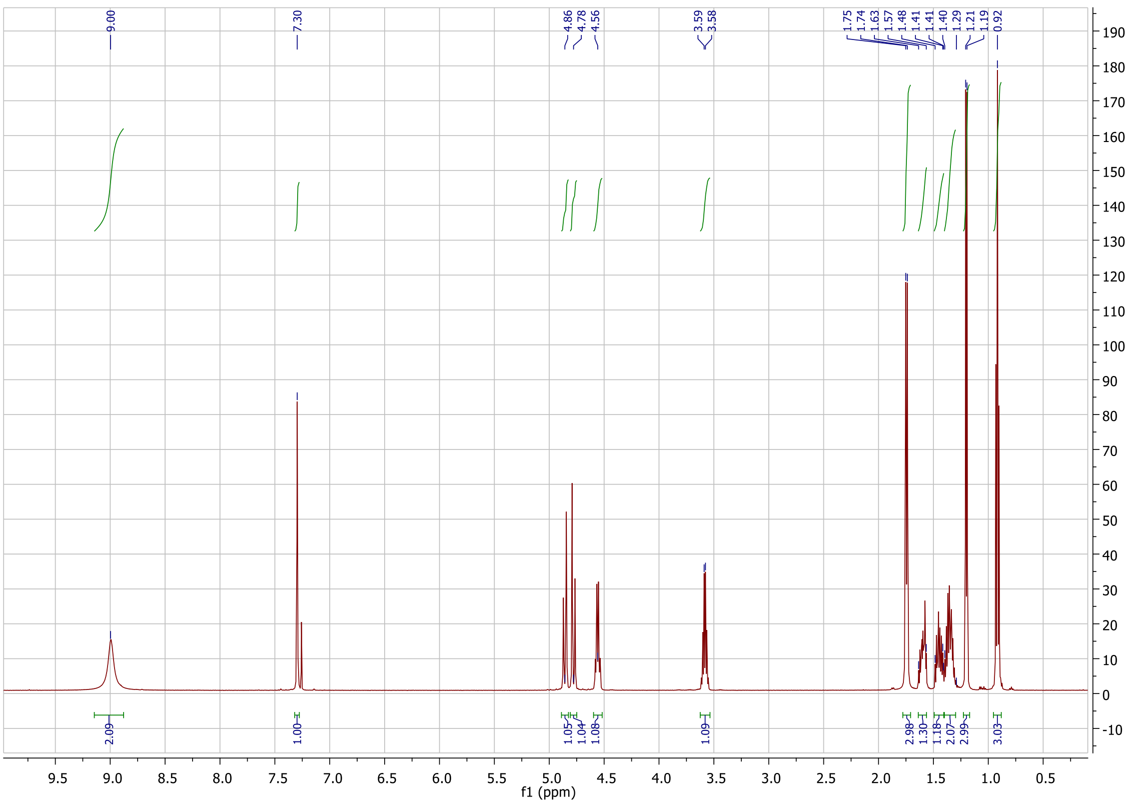

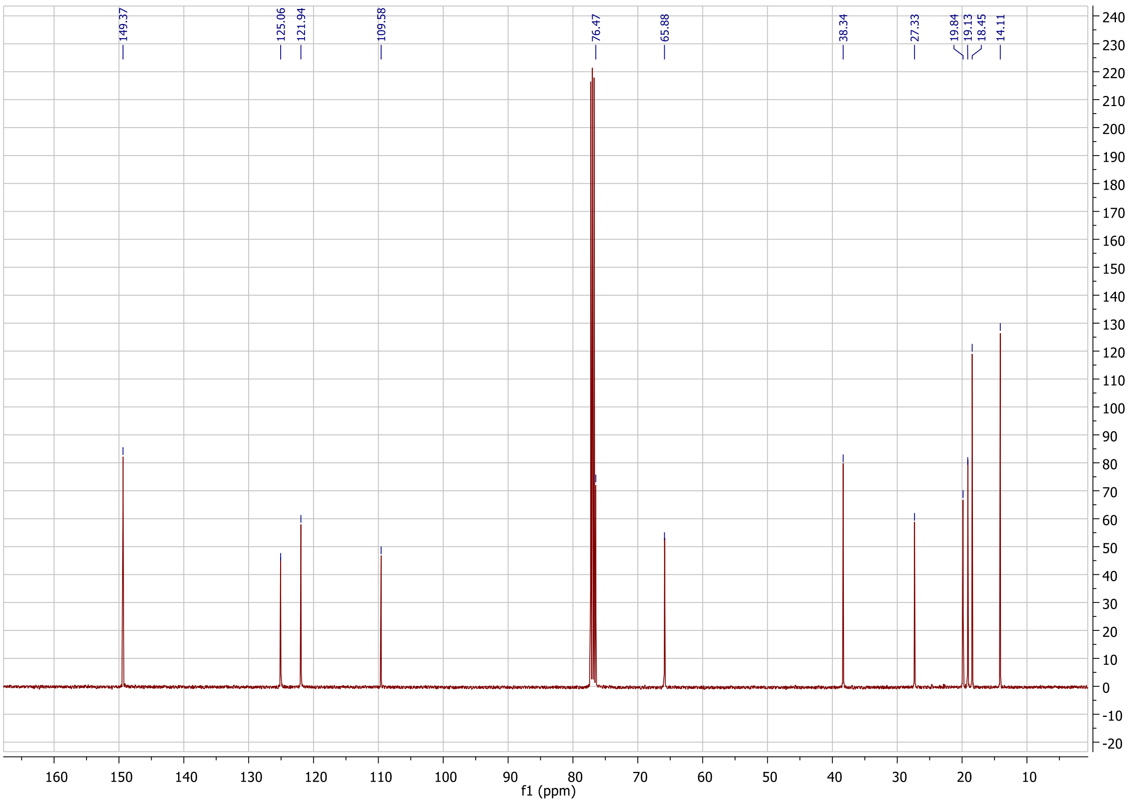

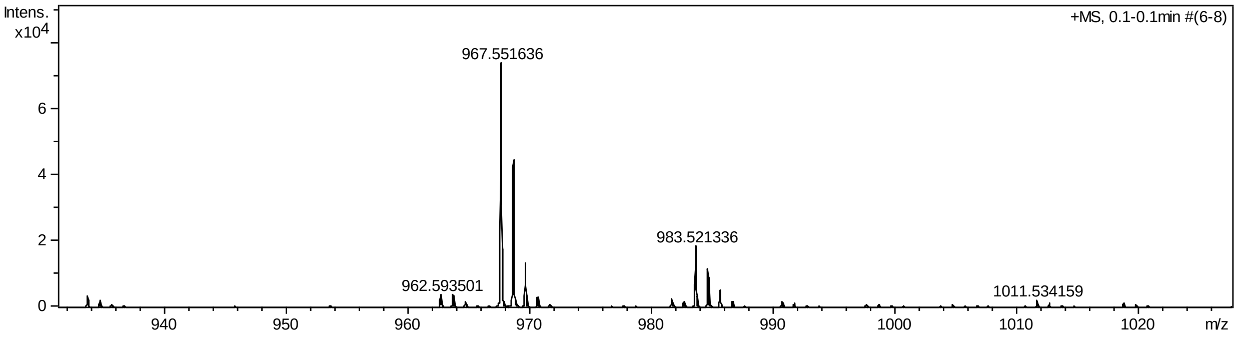
Intens.
x10^4
+MS, 0.1-0.1min #(6-8)
967.551636
983.521336
962.593501
1011.534159
m/z

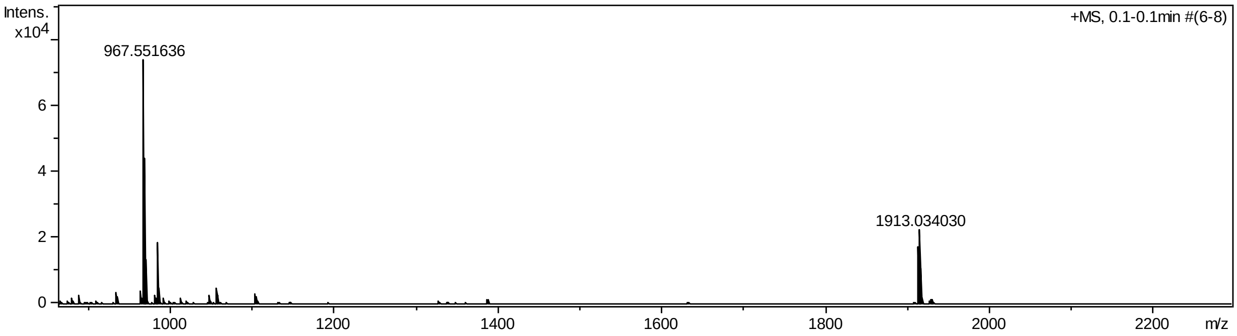
Intens.
x10^4
+MS, 0.1-0.1min #(6-8)
967.551636
1913.034030
m/z

tetra sec-hexoxy resorcinarene (2d). The crude product was purified by column chromatography to yield white solid product (0.23g, 63% yield); R_f = 0.80, (ethyl acetate/hexane, 1:4); mp > 300 °C; ^{1}H NMR (600 MHz, DMSO-d_6) δ (ppm): 8.52 (s, 8H, ArOH), 7.37 (s, 4H, ArH), 4.61 (d, *J* = 11.3 Hz, 4H, ArCH$_2$O), 4.51 (d, *J* = 11.4 Hz, 4H, ArCH$_2$O), 4.48 (q, *J* = 6.9 Hz, 4H, ArC*H*(CH$_3$)Ar), 3.50 (m, 4H, CH$_3$C*H*CH$_2$(CH$_2$)$_2$CH$_3$), 1.65 (d, *J* = 7.1 Hz, 12H, ArCH(C*H*$_3$)Ar), 1.54-1.45 (m, 4H, CH$_3$CHC*H*$_2$(CH$_2$)$_2$CH$_3$), 1.41-1.33 (m, 4H, CH$_3$CHC*H*$_2$(CH$_2$)$_2$CH$_3$), 1.32-1.21 (m, 8H, CH$_3$CHCH$_2$(C*H*$_2$)$_2$CH$_3$), 1.11 (d, *J* = 6.1 Hz, 12H, C*H*$_3$CHCH$_2$(CH$_2$)$_2$CH$_3$), 0.87 (t, *J* = 6.4 Hz, 12H, CH$_3$CHCH$_2$(CH$_2$)$_2$C*H*$_3$); ^{13}C NMR (151 MHz, DMSO-d_6) δ (ppm): 148.90, 124.51, 123.08, 110.61, 73.92, 61.20, 34.89, 28.31, 26.25, 21.53, 19.79, 18.62, 13.27; HR-MS (ESI): *m/z* calculated for $C_{60}H_{88}O_{12}K$ [M+K]$^+$ 1039.5912528, found: 1039.592197.

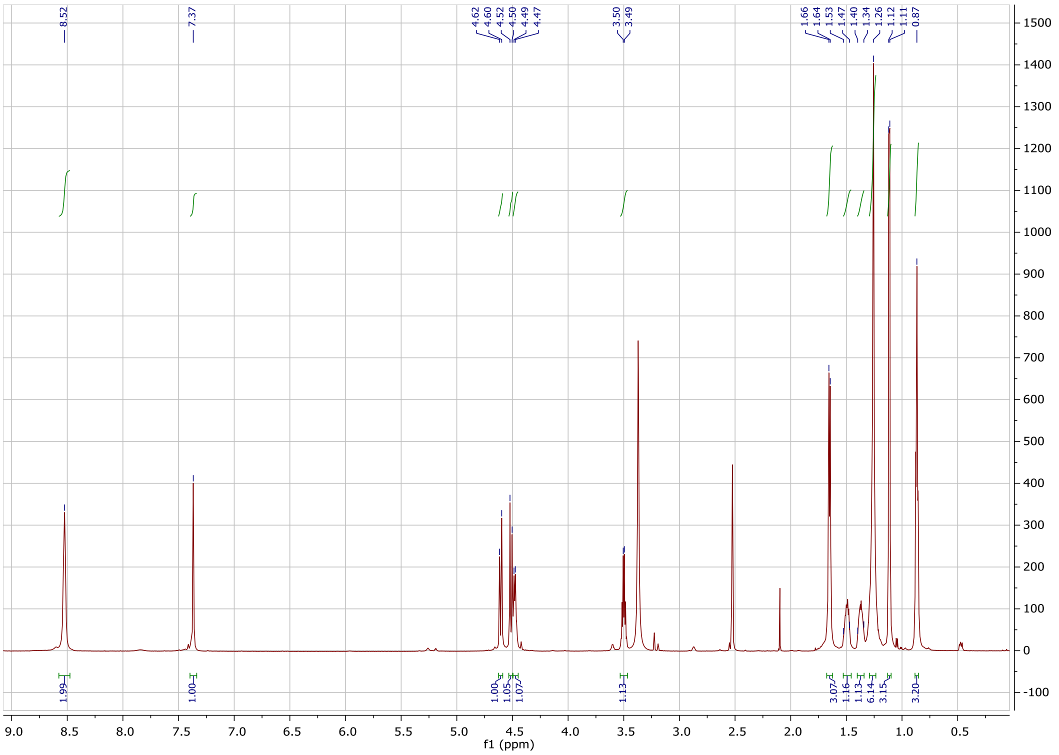

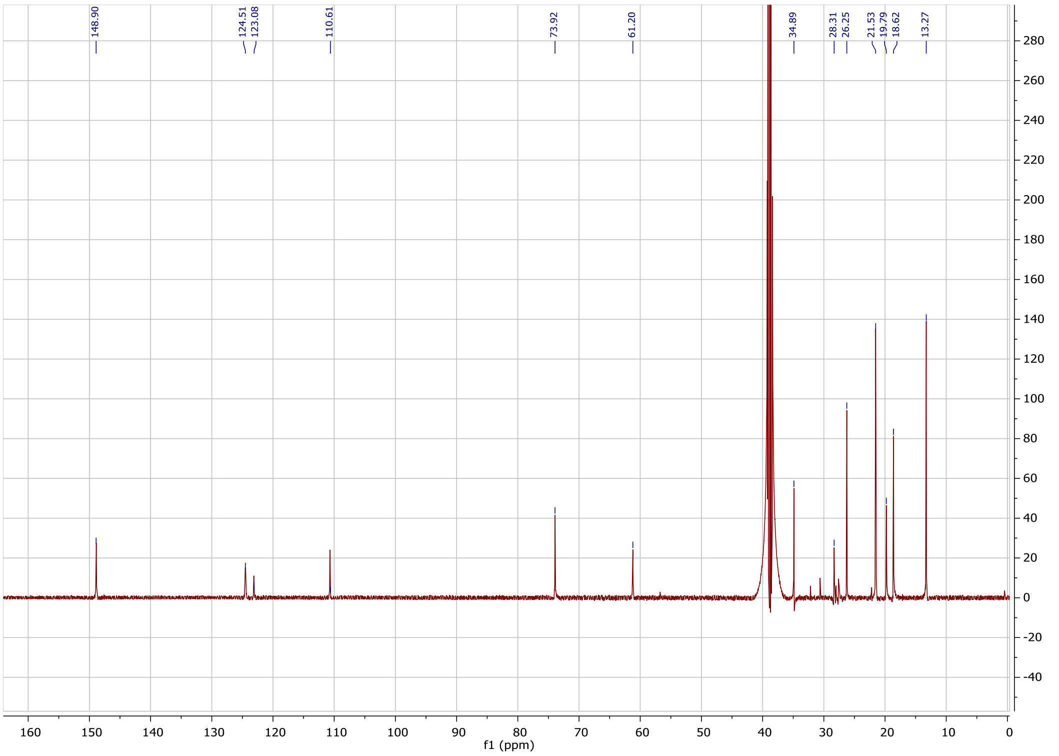

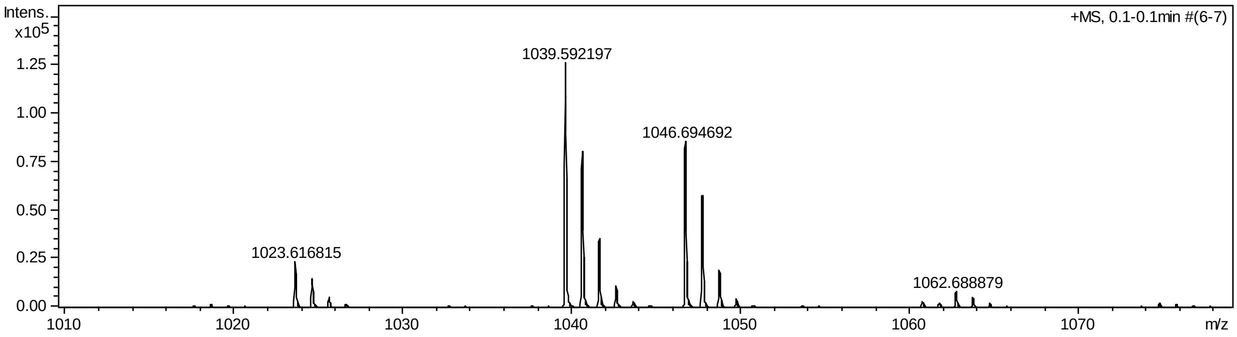
Intens.
x10^5
+MS, 0.1-0.1min #(6-7)
1039.592197
1046.694692
1023.616815
1062.688879
m/z

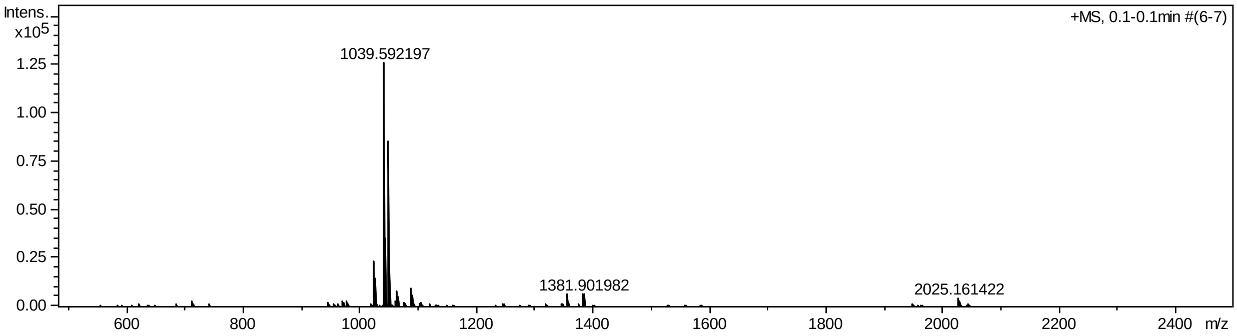
Intens.
x10^5
+MS, 0.1-0.1min #(6-7)
1039.592197
1381.901982
2025.161422
m/z

tetra cyclo-hexoxy resorcinarene (2e). The crude product was purified by column chromatography to yield white solid product (0.23g, 62% yield); R_f = 0.85, (ethyl acetate/hexane, 1:4); mp > 300 °C; ^1H NMR (500 MHz, DMSO-d_6) δ (ppm): 8.47 (s, 8H, ArOH), 7.29 (s, 4H, ArH), 4.54 (s, 8H, $ArCH_2O$), 4.44 (q, *J* = 7.1 Hz, 4H, Ar*CH*(CH_3)Ar), 3.38-3.33 (m, 4H, OC*H*$(CH_2)_2(CH_2)_2CH_2$), 1.86-1.80 (m, 8H, $OCH(CH_2)_2(CH_2)_2CH_2$), 1.59 (d, *J* = 7.2 Hz, 12H, $ArCH(CH_3)Ar$), 1.47-1.41 (m, 8H, $OCH(CH_2)_2(CH_2)_2CH_2$), 1.28-1.11 (m, 24H, $OCH(CH_2)_2(CH_2)_2CH_2$); ^{13}C NMR (126 MHz, DMSO-d_6) δ (ppm): 150.07, 125.59, 124.25, 111.77, 76.89, 62.01, 31.90, 28.76, 25.76, 23.79, 20.97; HR-MS (ESI): *m/z* calculated for $C_{60}H_{84}O_{12}N$ $[M+NH_4]^+$ 1010.5993204, found 1010.590201.

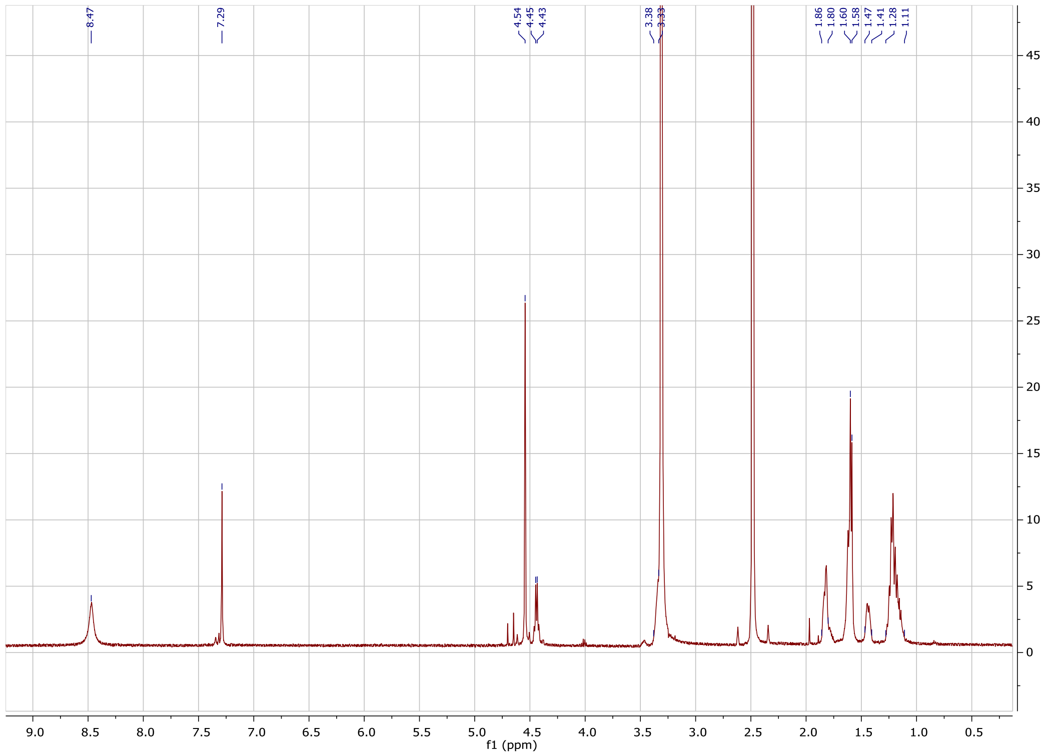

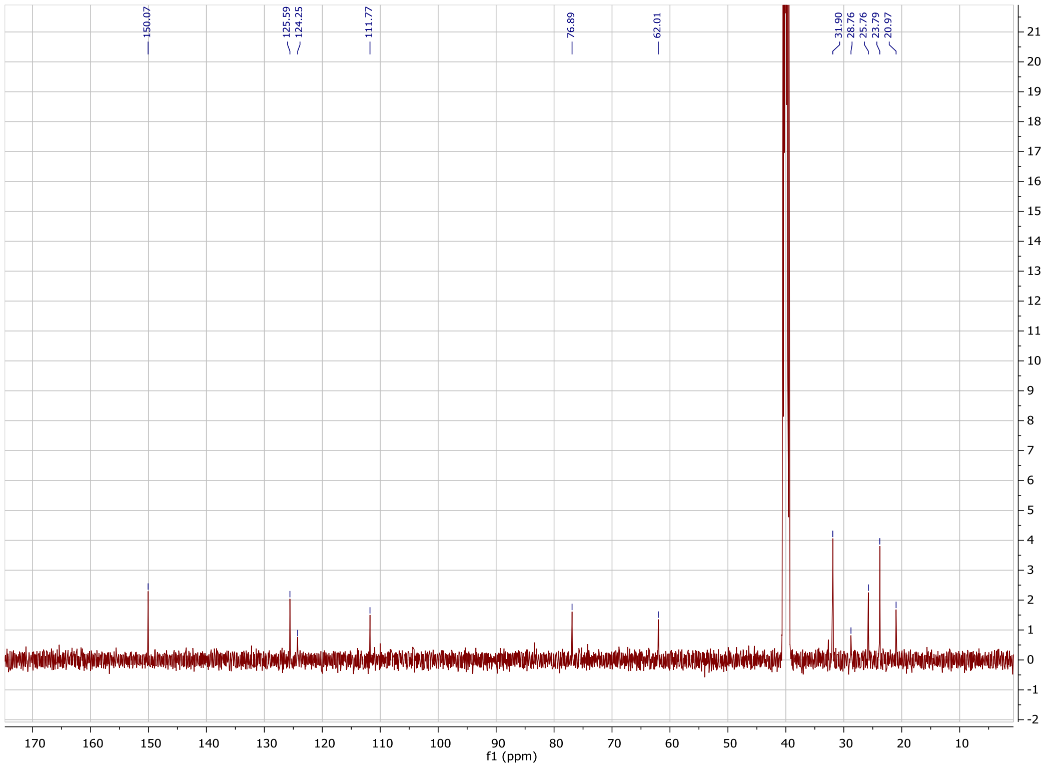

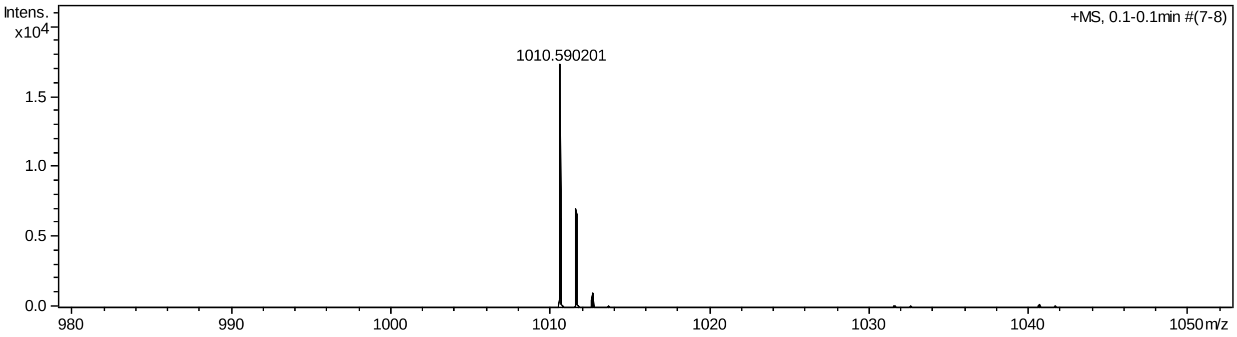
Intens.
x10^4
+MS, 0.1-0.1min #(7-8)
1010.590201
1.5
1.0
0.5
0.0
980
990
1000
1010
1020
1030
1040
1050
m/z

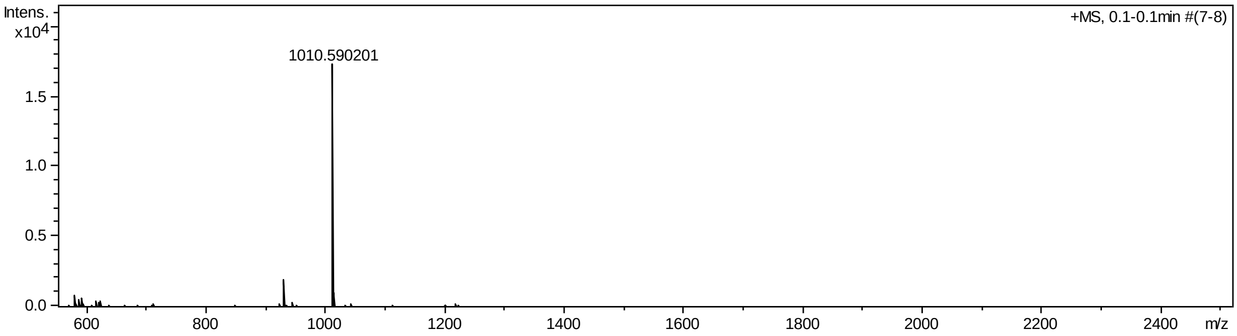
Intens.
x10^4
+MS, 0.1-0.1min #(7-8)
1010.590201
1.5
1.0
0.5
0.0
600
800
1000
1200
1400
1600
1800
2000
2200
2400
m/z

tetra tert-butyloxy resorcinarene (2f). The crude product was purified by column chromatography to yield white solid product (0.12g, 36% yield); R_f = 0.53 (ethyl acetate/hexane, 1:4); mp > 300 °C; ^{1}H NMR (500 MHz, $CDCl_3$) δ (ppm): 9.16 (s, 8H, ArOH), 7.27 (s, 4H, ArH), 4.77 (s, 8H, $ArCH_2O$), 4.54 (q, *J* = 7.4 Hz, 4H, ArC*H*(CH_3)Ar), 1.72 (d, *J* = 7.8 Hz, 12H, ArCH(C*H*$_3$)Ar), 1.29 (s, 36H, $OC(CH_3)_3$); ^{13}C NMR (126 MHz, $CDCl_3$) δ (ppm): 149.26, 125.06, 121.69, 110.02, 75.73, 60.45, 29.68, 27.29, 19.88; HR-MS (ESI): *m/z* calculated for $C_{52}H_{72}O_{12}Na$ $[M+Na]^+$ 911.4921212, found: 911.492667.

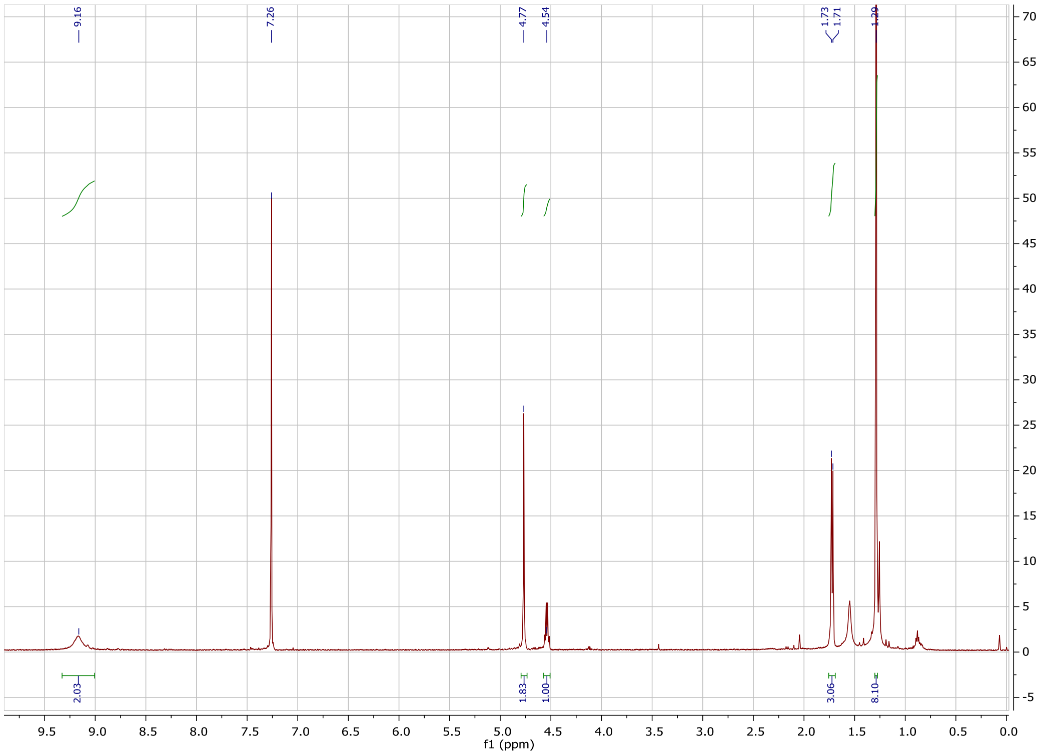

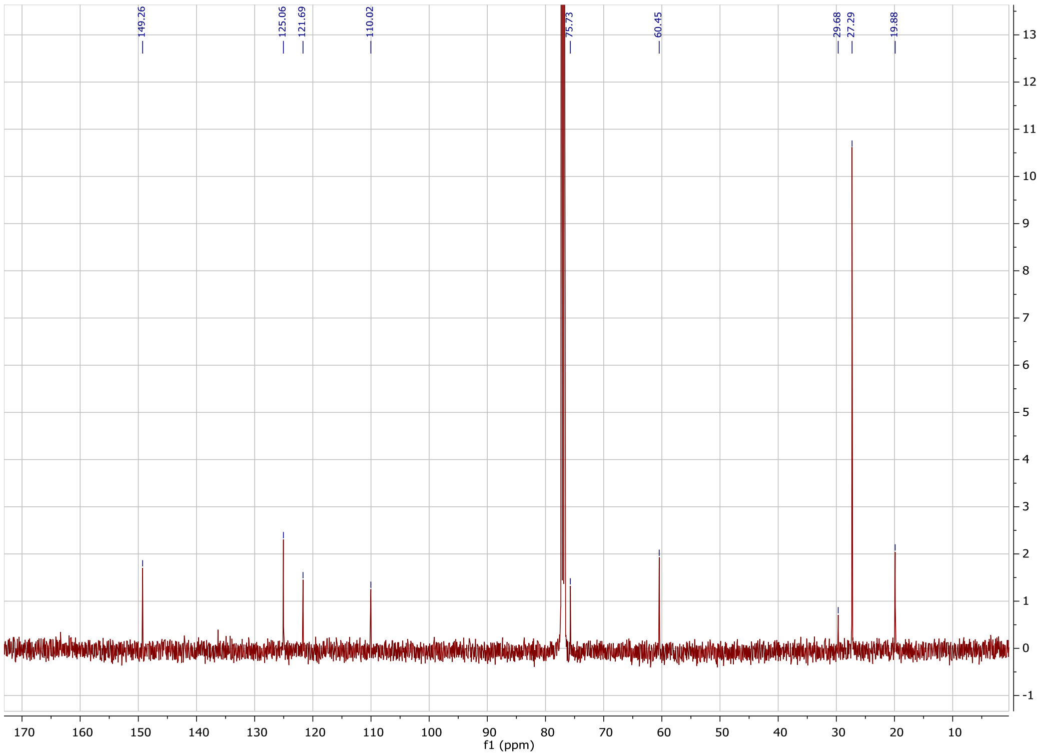

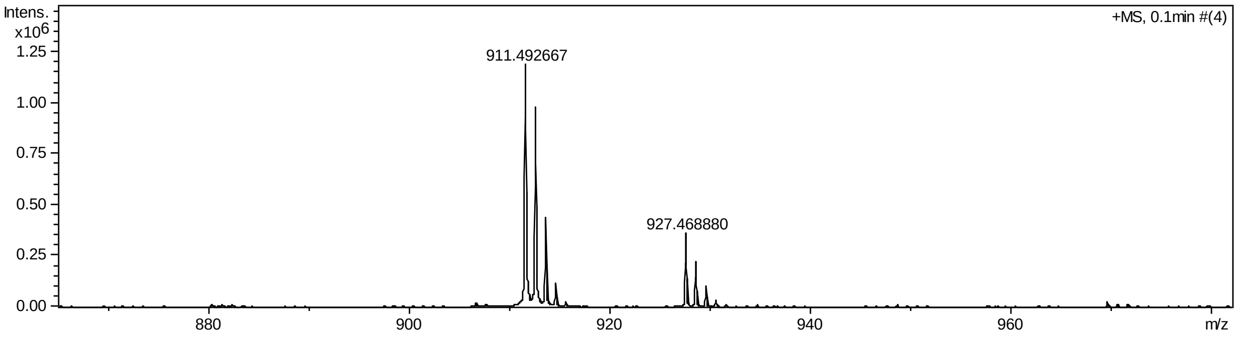
Intens.
x10^6
+MS, 0.1min #(4)
911.492667
927.468880
1.25
1.00
0.75
0.50
0.25
0.00
880
900
920
940
960
m/z

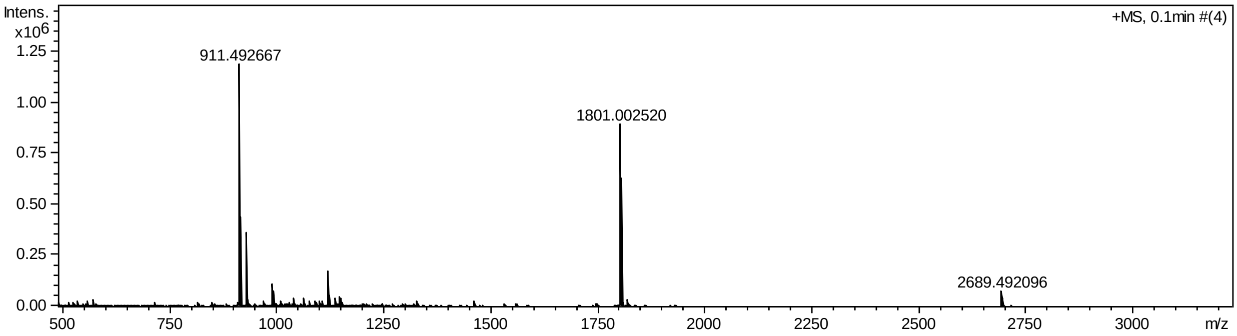
Intens.
x10^6
+MS, 0.1min #(4)
911.492667
1801.002520
2689.492096
1.25
1.00
0.75
0.50
0.25
0.00
500
750
1000
1250
1500
1750
2000
2250
2500
2750
3000
m/z

tetra tert-amyloxy resorcinarene (2g). The crude product was purified by column chromatography to yield white solid product (0.10g, 29% yield); R_f = 0.85 (ethyl acetate/hexane, 1:4); mp > 300 °C; ^1H NMR (500 MHz, $CDCl_3$) δ (ppm): 9.23 (s, 8H, ArOH), 7.27 (s, 4H, ArH), 4.74 (s, 8H, $ArCH_2O$), 4.54 (q, *J* = 7.4 Hz, 4H, $ArCH(CH_3)Ar$), 1.73 (d, *J* = 7.8 Hz, 12H, $ArCH(CH_3)Ar$), 1.60 (q, *J* = 7.9 Hz, 8H, $O(CH_3)_2CH_2CH_3$), 1.23 (s, 24H, $O(CH_3)_2CH_2CH_3$), 0.89 (q, *J* = 7.6 Hz, 12H, $O(CH_3)_2CH_2CH_3$); ^{13}C NMR (126 MHz, $CDCl_3$) δ (ppm): 149.28, 125.05, 121.64, 109.98, 77.92, 60.12, 32.70, 27.31, 24.66, 19.86, 8.33; HR-MS (ESI): *m/z* calculated for $C_{56}H_{80}O_{12}Na$ $[M+Na]^+$ 967.554718, found: 967.554282.

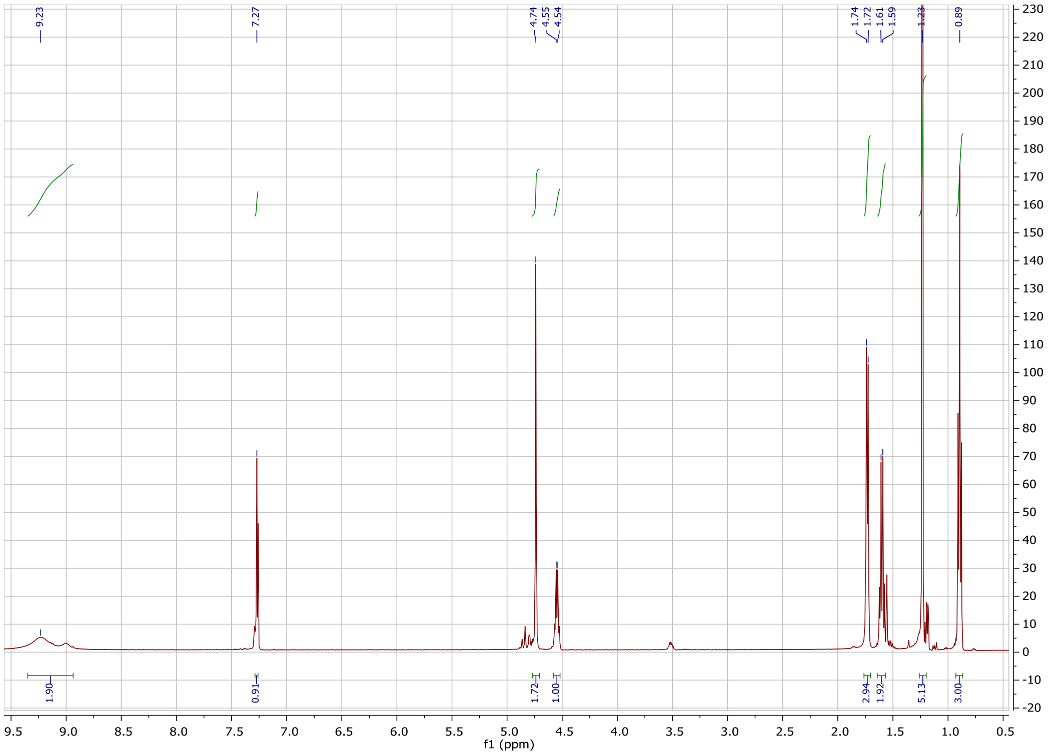

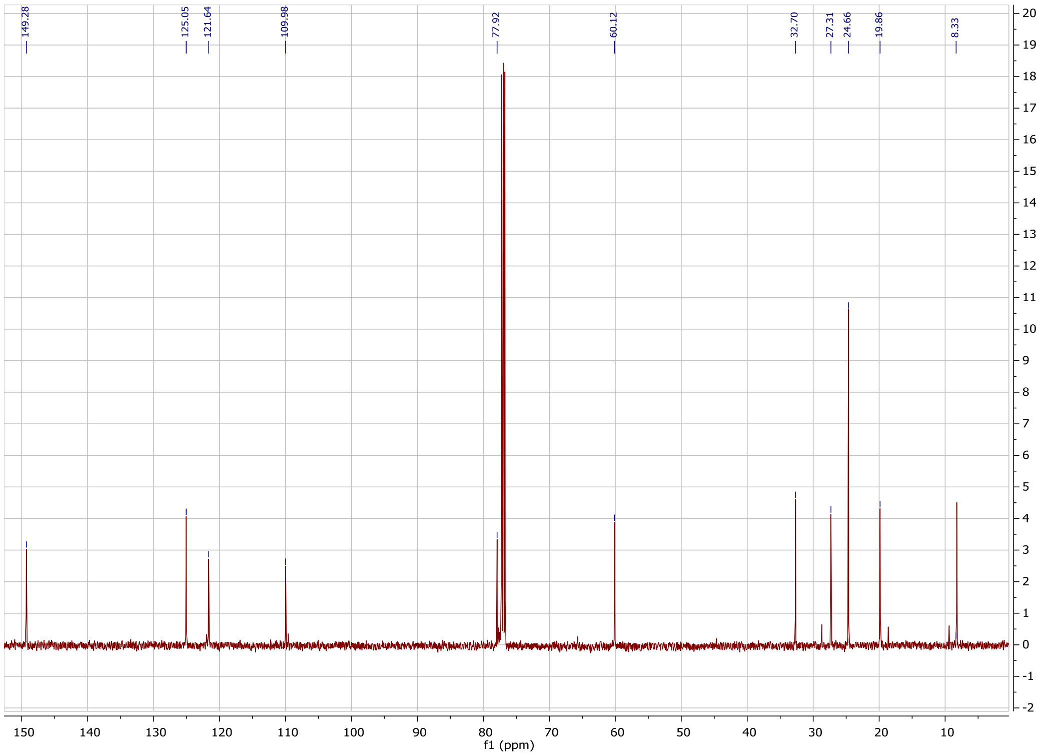

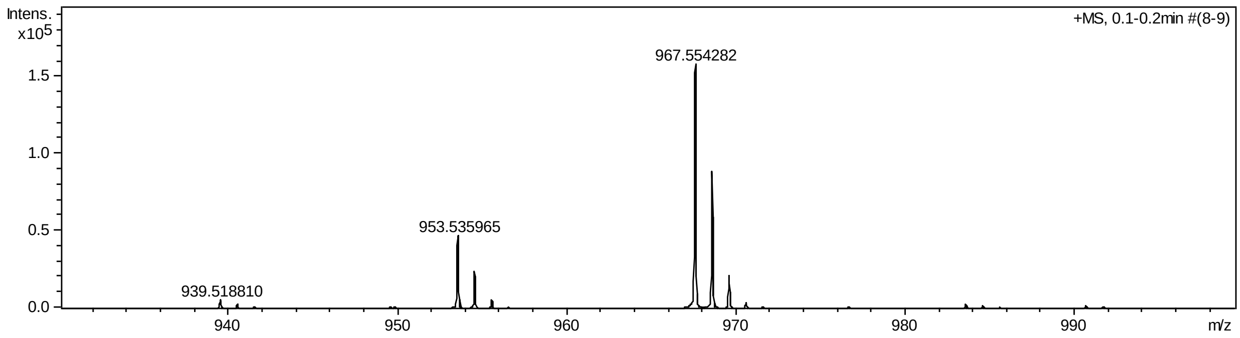
Intens.
x10^5
+MS, 0.1-0.2min #(8-9)
967.554282
953.535965
939.518810
m/z

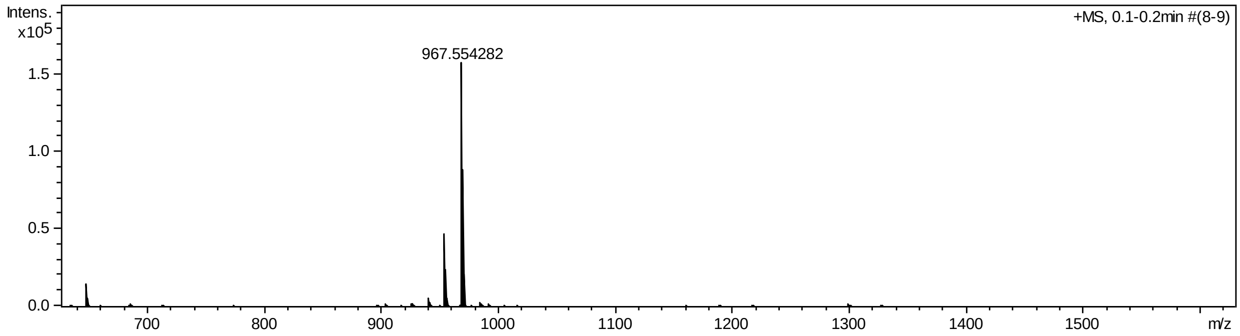
Intens.
x10^5
+MS, 0.1-0.2min #(8-9)
967.554282
m/z

Single-crystal X-ray diffraction data for compound 2a

Diffraction intensity data for the single crystal of the new compound **2a** were collected at 115 K on a Rigaku XtaLAB Synergy-S diffractometer with mirror-monochromated Mo Kα radiation (λ = 0.71073 Å). Cell refinement and data reduction were performed using firmware. The positions of all non-hydrogen atoms were determined by direct methods using SHELXT 2014/4 software. All non-hydrogen atoms were refined anisotropically using weighted full-matrix least-squares on F2. Refinement and further calculations were carried out using SHELXL software (ver. 2019/2)

All hydrogen atoms joined to carbon atoms were positioned with idealized geometries and refined using a riding model with Uiso(H) fixed at 1.2 Ueq (Carom). The figures were made using Diamond ver. 4.6.1 software. CCDC 2297228 contains the supplementary crystallographic data for **2a**.

Table S1. Bond lengths [Å] and angles [°] for **2a**.

O(1)-C(6)	1.3811(17)	O(3)-C(13)-C(8)	117.88(12)
O(1)-H(1OO)	0.84	O(3)-C(13)-C(12)	119.65(13)
O(3)-C(13)	1.3864(17)	C(8)-C(13)-C(12)	122.46(13)
O(3)-H(3O)	0.84	O(4)-C(11)-C(12)	116.12(13)
O(7)-C(27)	1.3817(17)	O(4)-C(11)-C(10)	122.65(13)
O(7)-H(7O)	0.84	C(12)-C(11)-C(10)	121.22(13)
O(6)-C(18)	1.3762(18)	O(1)-C(6)-C(5)	121.47(12)
O(6)-H(6O)	0.84	O(1)-C(6)-C(1)	116.31(13)
O(13)-C(34)	1.438(2)	C(5)-C(6)-C(1)	122.22(13)
O(13)-C(33)	1.4412(18)	O(6)-C(18)-C(19)	116.19(13)
O(2)-C(4)	1.3682(18)	O(6)-C(18)-C(17)	122.37(13)
O(2)-H(2O)	0.84	C(19)-C(18)-C(17)	121.41(13)
O(5)-C(20)	1.3853(18)	C(17)-C(21)-C(22)	111.16(11)
O(5)-H(5O)	0.84	C(17)-C(21)-C(32)	112.51(12)
O(8)-C(25)	1.3723(17)	C(22)-C(21)-C(32)	112.36(12)
O(8)-H(8O)	0.84	C(17)-C(21)-H(21)	106.8
O(4)-C(11)	1.3718(17)	C(22)-C(21)-H(21)	106.8
O(4)-H(4O)	0.84	C(32)-C(21)-H(21)	106.8
O(88)-C(100)	1.331(3)	C(9)-C(8)-C(13)	116.80(13)
O(88)-C(101)	1.450(3)	C(9)-C(8)-C(7)	122.68(13)
O(10)-C(38B)	1.213(4)	C(13)-C(8)-C(7)	120.52(12)
O(10)-C(38A)	1.336(3)	C(27)-C(26)-C(25)	118.33(13)
O(10)-C(37)	1.438(2)	C(27)-C(26)-C(45)	122.66(13)
O(12)-C(100)	1.212(2)	C(25)-C(26)-C(45)	119.00(13)
O(12A)-C(46A)	1.453(7)	C(9)-C(10)-C(11)	117.62(13)
O(12A)-C(45)	1.496(3)	C(9)-C(10)-C(14)	121.76(13)
C(46A)-C(48A)	1.509(7)	C(11)-C(10)-C(14)	120.63(13)
C(46A)-C(47A)	1.678(15)	C(16)-C(17)-C(18)	117.56(13)
C(46A)-H(46A)	1	C(16)-C(17)-C(21)	121.02(13)
C(47A)-H(47A)	0.98	C(18)-C(17)-C(21)	121.42(13)
C(47A)-H(47B)	0.98	C(15)-C(16)-C(17)	123.11(13)
C(47A)-H(47C)	0.98	C(15)-C(16)-H(16)	118.4
C(48A)-H(48A)	0.98	C(17)-C(16)-H(16)	118.4
C(48A)-H(48B)	0.98	O(7)-C(27)-C(26)	120.34(12)
C(48A)-H(48C)	0.98	O(7)-C(27)-C(22)	117.47(12)
O(12B)-C(45)	1.333(4)	C(26)-C(27)-C(22)	122.19(13)
O(12B)-C(46B)	1.453(9)	C(1)-C(28)-C(24)	111.70(11)
C(46B)-C(47B)	1.143(17)	C(1)-C(28)-C(29)	111.96(11)
C(46B)-C(48B)	1.524(9)	C(24)-C(28)-C(29)	111.99(11)
C(46B)-H(46B)	1	C(1)-C(28)-H(28)	106.9
C(47B)-H(47D)	0.98	C(24)-C(28)-H(28)	106.9
C(47B)-H(47E)	0.98	C(29)-C(28)-H(28)	106.9
C(47B)-H(47F)	0.98	C(23)-C(22)-C(27)	117.26(13)

C(48B)-H(48D)	0.98	C(23)-C(22)-C(21)	122.25(12)
C(48B)-H(48E)	0.98	C(27)-C(22)-C(21)	120.49(12)
C(48B)-H(48F)	0.98	O(2)-C(4)-C(5)	116.28(12)
C(3)-C(2)	1.387(2)	O(2)-C(4)-C(3)	122.64(13)
C(3)-C(4)	1.4029(19)	C(5)-C(4)-C(3)	121.07(14)
C(3)-C(7)	1.5280(19)	C(18)-C(19)-C(20)	118.65(13)
C(24)-C(23)	1.3918(19)	C(18)-C(19)-C(41)	120.07(13)
C(24)-C(25)	1.403(2)	C(20)-C(19)-C(41)	121.28(14)
C(24)-C(28)	1.5295(19)	C(6)-C(5)-C(4)	118.63(13)
C(1)-C(2)	1.3925(19)	C(6)-C(5)-C(33)	120.49(13)
C(1)-C(6)	1.3965(19)	C(4)-C(5)-C(33)	120.87(14)
C(1)-C(28)	1.524(2)	O(8)-C(25)-C(26)	115.82(12)
C(12)-C(13)	1.397(2)	O(8)-C(25)-C(24)	122.66(13)
C(12)-C(11)	1.397(2)	C(26)-C(25)-C(24)	121.50(13)
C(12)-C(37)	1.503(2)	C(16)-C(15)-C(20)	117.28(13)
C(23)-C(22)	1.3886(19)	C(16)-C(15)-C(14)	122.11(13)
C(23)-H(23)	0.95	C(20)-C(15)-C(14)	120.60(13)
C(20)-C(19)	1.394(2)	C(8)-C(9)-C(10)	123.41(13)
C(20)-C(15)	1.396(2)	C(8)-C(9)-H(9)	118.3
C(13)-C(8)	1.395(2)	C(10)-C(9)-H(9)	118.3
C(11)-C(10)	1.402(2)	C(7)-C(30)-H(30A)	109.5
C(6)-C(5)	1.390(2)	C(7)-C(30)-H(30B)	109.5
C(18)-C(19)	1.392(2)	H(30A)-C(30)-H(30B)	109.5
C(18)-C(17)	1.400(2)	C(7)-C(30)-H(30C)	109.5
C(21)-C(17)	1.523(2)	H(30A)-C(30)-H(30C)	109.5
C(21)-C(22)	1.5266(19)	H(30B)-C(30)-H(30C)	109.5
C(21)-C(32)	1.535(2)	C(8)-C(7)-C(3)	111.54(11)
C(21)-H(21)	1	C(8)-C(7)-C(30)	112.25(11)
C(8)-C(9)	1.3915(19)	C(3)-C(7)-C(30)	112.31(12)
C(8)-C(7)	1.5236(19)	C(8)-C(7)-H(7)	106.8
C(26)-C(27)	1.393(2)	C(3)-C(7)-H(7)	106.8
C(26)-C(25)	1.399(2)	C(30)-C(7)-H(7)	106.8
C(26)-C(45)	1.508(2)	C(28)-C(29)-H(29A)	109.5
C(10)-C(9)	1.394(2)	C(28)-C(29)-H(29B)	109.5
C(10)-C(14)	1.5271(19)	H(29A)-C(29)-H(29B)	109.5
C(17)-C(16)	1.394(2)	C(28)-C(29)-H(29C)	109.5
C(16)-C(15)	1.388(2)	H(29A)-C(29)-H(29C)	109.5
C(16)-H(16)	0.95	H(29B)-C(29)-H(29C)	109.5
C(27)-C(22)	1.395(2)	C(15)-C(14)-C(10)	112.22(11)
C(28)-C(29)	1.5348(19)	C(15)-C(14)-C(31)	111.31(12)
C(28)-H(28)	1	C(10)-C(14)-C(31)	112.78(12)
C(4)-C(5)	1.401(2)	C(15)-C(14)-H(14)	106.7
C(19)-C(41)	1.510(2)	C(10)-C(14)-H(14)	106.7
C(5)-C(33)	1.5083(19)	C(31)-C(14)-H(14)	106.7
C(15)-C(14)	1.523(2)	O(13)-C(33)-C(5)	111.57(12)
C(9)-H(9)	0.95	O(13)-C(33)-H(33A)	109.3
C(30)-C(7)	1.5379(19)	C(5)-C(33)-H(33A)	109.3
C(30)-H(30A)	0.98	O(13)-C(33)-H(33B)	109.3
C(30)-H(30B)	0.98	C(5)-C(33)-H(33B)	109.3
C(30)-H(30C)	0.98	H(33A)-C(33)-H(33B)	108
C(7)-H(7)	1	C(3)-C(2)-C(1)	123.46(12)
C(29)-H(29A)	0.98	C(3)-C(2)-H(2)	118.3
C(29)-H(29B)	0.98	C(1)-C(2)-H(2)	118.3
C(29)-H(29C)	0.98	C(14)-C(31)-H(31A)	109.5
C(14)-C(31)	1.532(2)	C(14)-C(31)-H(31B)	109.5
C(14)-H(14)	1	H(31A)-C(31)-H(31B)	109.5
C(33)-H(33A)	0.99	C(14)-C(31)-H(31C)	109.5
C(33)-H(33B)	0.99	H(31A)-C(31)-H(31C)	109.5
C(2)-H(2)	0.95	H(31B)-C(31)-H(31C)	109.5
C(31)-H(31A)	0.98	C(21)-C(32)-H(32A)	109.5

C(31)-H(31B)	0.98
C(31)-H(31C)	0.98
C(32)-H(32A)	0.98
C(32)-H(32B)	0.98
C(32)-H(32C)	0.98
C(45)-H(45A)	0.99
C(45)-H(45B)	0.99
C(41)-O(11B)	1.313(5)
C(41)-O(11A)	1.524(3)
C(41)-H(41A)	0.99
C(41)-H(41B)	0.99
C(37)-H(37A)	0.99
C(37)-H(37B)	0.99
C(34)-C(36)	1.500(3)
C(34)-C(35)	1.506(3)
C(34)-H(34)	1
C(100)-C(99)	1.483(3)
C(36)-H(36A)	0.98
C(36)-H(36B)	0.98
C(36)-H(36C)	0.98
C(99)-H(99A)	0.98
C(99)-H(99B)	0.98
C(99)-H(99C)	0.98
C(101)-C(102)	1.467(3)
C(101)-H(10A)	0.99
C(101)-H(10B)	0.99
C(102)-H(10C)	0.98
C(102)-H(10D)	0.98
C(102)-H(10E)	0.98
C(35)-H(35A)	0.98
C(35)-H(35B)	0.98
C(35)-H(35C)	0.98
C(38A)-C(40A)	1.487(5)
C(38A)-C(39A)	1.503(4)
C(38A)-H(38A)	1
C(40A)-H(40A)	0.98
C(40A)-H(40B)	0.98
C(40A)-H(40C)	0.98
C(39A)-H(39A)	0.98
C(39A)-H(39B)	0.98
C(39A)-H(39C)	0.98
C(38B)-C(40B)	1.490(6)
C(38B)-C(39B)	1.491(8)
C(38B)-H(38B)	1
C(40B)-H(40D)	0.98
C(40B)-H(40E)	0.98
C(40B)-H(40F)	0.98
C(39B)-H(39D)	0.98
C(39B)-H(39E)	0.98
C(39B)-H(39F)	0.98
O(11A)-C(42A)	1.437(4)
C(42A)-C(44A)	1.491(5)
C(42A)-C(43A)	1.524(5)
C(42A)-H(42A)	1
C(44A)-H(44A)	0.98
C(44A)-H(44B)	0.98
C(44A)-H(44C)	0.98
C(43A)-H(43A)	0.98
C(43A)-H(43B)	0.98
C(43A)-H(43C)	0.98

C(21)-C(32)-H(32B)	109.5
H(32A)-C(32)-H(32B)	109.5
C(21)-C(32)-H(32C)	109.5
H(32A)-C(32)-H(32C)	109.5
H(32B)-C(32)-H(32C)	109.5
O(12B)-C(45)-C(26)	114.80(19)
O(12A)-C(45)-C(26)	106.87(16)
O(12A)-C(45)-H(45A)	110.3
C(26)-C(45)-H(45A)	110.3
O(12A)-C(45)-H(45B)	110.3
C(26)-C(45)-H(45B)	110.3
H(45A)-C(45)-H(45B)	108.6
O(11B)-C(41)-C(19)	116.5(2)
C(19)-C(41)-O(11A)	103.68(16)
C(19)-C(41)-H(41A)	111
O(11A)-C(41)-H(41A)	111
C(19)-C(41)-H(41B)	111
O(11A)-C(41)-H(41B)	111
H(41A)-C(41)-H(41B)	109
O(10)-C(37)-C(12)	110.25(14)
O(10)-C(37)-H(37A)	109.6
C(12)-C(37)-H(37A)	109.6
O(10)-C(37)-H(37B)	109.6
C(12)-C(37)-H(37B)	109.6
H(37A)-C(37)-H(37B)	108.1
O(13)-C(34)-C(36)	105.70(15)
O(13)-C(34)-C(35)	110.44(16)
C(36)-C(34)-C(35)	113.90(18)
O(13)-C(34)-H(34)	108.9
C(36)-C(34)-H(34)	108.9
C(35)-C(34)-H(34)	108.9
O(12)-C(100)-O(88)	124.2(2)
O(12)-C(100)-C(99)	125.2(2)
O(88)-C(100)-C(99)	110.56(17)
C(34)-C(36)-H(36A)	109.5
C(34)-C(36)-H(36B)	109.5
H(36A)-C(36)-H(36B)	109.5
C(34)-C(36)-H(36C)	109.5
H(36A)-C(36)-H(36C)	109.5
H(36B)-C(36)-H(36C)	109.5
C(100)-C(99)-H(99A)	109.5
C(100)-C(99)-H(99B)	109.5
H(99A)-C(99)-H(99B)	109.5
C(100)-C(99)-H(99C)	109.5
H(99A)-C(99)-H(99C)	109.5
H(99B)-C(99)-H(99C)	109.5
O(88)-C(101)-C(102)	107.75(17)
O(88)-C(101)-H(10A)	110.2
C(102)-C(101)-H(10A)	110.2
O(88)-C(101)-H(10B)	110.2
C(102)-C(101)-H(10B)	110.2
H(10A)-C(101)-H(10B)	108.5
C(101)-C(102)-H(10C)	109.5
C(101)-C(102)-H(10D)	109.5
H(10C)-C(102)-H(10D)	109.5
C(101)-C(102)-H(10E)	109.5
H(10C)-C(102)-H(10E)	109.5
H(10D)-C(102)-H(10E)	109.5
C(34)-C(35)-H(35A)	109.5
C(34)-C(35)-H(35B)	109.5

O(11B)-C(42B)	1.457(17)
C(42B)-C(43B)	1.496(15)
C(42B)-C(44B)	1.571(19)
C(42B)-H(42B)	1
C(44B)-H(44D)	0.98
C(44B)-H(44E)	0.98
C(44B)-H(44F)	0.98
C(43B)-H(43D)	0.98
C(43B)-H(43E)	0.98
C(43B)-H(43F)	0.98
C(6)-O(1)-H(1OO)	109.5
C(13)-O(3)-H(3O)	109.5
C(27)-O(7)-H(7O)	109.5
C(18)-O(6)-H(6O)	109.5
C(34)-O(13)-C(33)	113.72(11)
C(4)-O(2)-H(2O)	109.5
C(20)-O(5)-H(5O)	109.5
C(25)-O(8)-H(8O)	109.5
C(11)-O(4)-H(4O)	109.5
C(100)-O(88)-C(101)	116.84(16)
C(38B)-O(10)-C(37)	119.7(3)
C(38A)-O(10)-C(37)	124.90(19)
C(46A)-O(12A)-C(45)	112.7(3)
O(12A)-C(46A)-C(48A)	105.9(4)
O(12A)-C(46A)-C(47A)	114.3(6)
C(48A)-C(46A)-C(47A)	97.3(5)
O(12A)-C(46A)-H(46A)	112.7
C(48A)-C(46A)-H(46A)	112.7
C(47A)-C(46A)-H(46A)	112.7
C(46A)-C(47A)-H(47A)	109.5
C(46A)-C(47A)-H(47B)	109.5
H(47A)-C(47A)-H(47B)	109.5
C(46A)-C(47A)-H(47C)	109.5
H(47A)-C(47A)-H(47C)	109.5
H(47B)-C(47A)-H(47C)	109.5
C(46A)-C(48A)-H(48A)	109.5
C(46A)-C(48A)-H(48B)	109.5
H(48A)-C(48A)-H(48B)	109.5
C(46A)-C(48A)-H(48C)	109.5
H(48A)-C(48A)-H(48C)	109.5
H(48B)-C(48A)-H(48C)	109.5
C(45)-O(12B)-C(46B)	116.1(4)
C(47B)-C(46B)-O(12B)	105.4(11)
C(47B)-C(46B)-C(48B)	130.0(10)
O(12B)-C(46B)-C(48B)	108.3(5)
C(47B)-C(46B)-H(46B)	103.4
O(12B)-C(46B)-H(46B)	103.4
C(48B)-C(46B)-H(46B)	103.4
C(46B)-C(47B)-H(47D)	109.5
C(46B)-C(47B)-H(47E)	109.5
H(47D)-C(47B)-H(47E)	109.5
C(46B)-C(47B)-H(47F)	109.5
H(47D)-C(47B)-H(47F)	109.5
H(47E)-C(47B)-H(47F)	109.5
C(46B)-C(48B)-H(48D)	109.5
C(46B)-C(48B)-H(48E)	109.5
H(48D)-C(48B)-H(48E)	109.5
C(46B)-C(48B)-H(48F)	109.5
H(48D)-C(48B)-H(48F)	109.5
H(48E)-C(48B)-H(48F)	109.5
H(35A)-C(35)-H(35B)	109.5
C(34)-C(35)-H(35C)	109.5
H(35A)-C(35)-H(35C)	109.5
H(35B)-C(35)-H(35C)	109.5
O(10)-C(38A)-C(40A)	111.5(3)
O(10)-C(38A)-C(39A)	113.3(3)
C(40A)-C(38A)-C(39A)	113.1(3)
O(10)-C(38A)-H(38A)	106.1
C(40A)-C(38A)-H(38A)	106.1
C(39A)-C(38A)-H(38A)	106.1
C(38A)-C(40A)-H(40A)	109.5
C(38A)-C(40A)-H(40B)	109.5
H(40A)-C(40A)-H(40B)	109.5
C(38A)-C(40A)-H(40C)	109.5
H(40A)-C(40A)-H(40C)	109.5
H(40B)-C(40A)-H(40C)	109.5
C(38A)-C(39A)-H(39A)	109.5
C(38A)-C(39A)-H(39B)	109.5
H(39A)-C(39A)-H(39B)	109.5
C(38A)-C(39A)-H(39C)	109.5
H(39A)-C(39A)-H(39C)	109.5
H(39B)-C(39A)-H(39C)	109.5
O(10)-C(38B)-C(40B)	120.7(4)
O(10)-C(38B)-C(39B)	107.0(5)
C(40B)-C(38B)-C(39B)	111.9(4)
O(10)-C(38B)-H(38B)	105.3
C(40B)-C(38B)-H(38B)	105.3
C(39B)-C(38B)-H(38B)	105.3
C(38B)-C(40B)-H(40D)	109.5
C(38B)-C(40B)-H(40E)	109.5
H(40D)-C(40B)-H(40E)	109.5
C(38B)-C(40B)-H(40F)	109.5
H(40D)-C(40B)-H(40F)	109.5
H(40E)-C(40B)-H(40F)	109.5
C(38B)-C(39B)-H(39D)	109.5
C(38B)-C(39B)-H(39E)	109.5
H(39D)-C(39B)-H(39E)	109.5
C(38B)-C(39B)-H(39F)	109.5
H(39D)-C(39B)-H(39F)	109.5
H(39E)-C(39B)-H(39F)	109.5
C(42A)-O(11A)-C(41)	115.3(2)
O(11A)-C(42A)-C(44A)	113.8(3)
O(11A)-C(42A)-C(43A)	104.3(3)
C(44A)-C(42A)-C(43A)	110.6(3)
O(11A)-C(42A)-H(42A)	109.3
C(44A)-C(42A)-H(42A)	109.3
C(43A)-C(42A)-H(42A)	109.3
C(42A)-C(44A)-H(44A)	109.5
C(42A)-C(44A)-H(44B)	109.5
H(44A)-C(44A)-H(44B)	109.5
C(42A)-C(44A)-H(44C)	109.5
H(44A)-C(44A)-H(44C)	109.5
H(44B)-C(44A)-H(44C)	109.5
C(42A)-C(43A)-H(43A)	109.5
C(42A)-C(43A)-H(43B)	109.5
H(43A)-C(43A)-H(43B)	109.5
C(42A)-C(43A)-H(43C)	109.5
H(43A)-C(43A)-H(43C)	109.5
H(43B)-C(43A)-H(43C)	109.5
C(41)-O(11B)-C(42B)	115.0(7)

C(2)-C(3)-C(4)	117.58(13)	O(11B)-C(42B)-C(43B)	100.9(12)
C(2)-C(3)-C(7)	121.37(12)	O(11B)-C(42B)-C(44B)	91.6(16)
C(4)-C(3)-C(7)	121.04(13)	C(43B)-C(42B)-C(44B)	88.9(16)
C(23)-C(24)-C(25)	117.33(13)	O(11B)-C(42B)-H(42B)	122.4
C(23)-C(24)-C(28)	121.40(12)	C(43B)-C(42B)-H(42B)	122.4
C(25)-C(24)-C(28)	121.27(12)	C(44B)-C(42B)-H(42B)	122.4
C(2)-C(1)-C(6)	116.95(13)	C(42B)-C(44B)-H(44D)	109.5
C(2)-C(1)-C(28)	122.13(12)	C(42B)-C(44B)-H(44E)	109.5
C(6)-C(1)-C(28)	120.92(12)	H(44D)-C(44B)-H(44E)	109.5
C(13)-C(12)-C(11)	118.46(13)	C(42B)-C(44B)-H(44F)	109.5
C(13)-C(12)-C(37)	121.53(13)	H(44D)-C(44B)-H(44F)	109.5
C(11)-C(12)-C(37)	120.00(13)	H(44E)-C(44B)-H(44F)	109.5
C(22)-C(23)-C(24)	123.33(13)	C(42B)-C(43B)-H(43D)	109.5
C(22)-C(23)-H(23)	118.3	C(42B)-C(43B)-H(43E)	109.5
C(24)-C(23)-H(23)	118.3	H(43D)-C(43B)-H(43E)	109.5
O(5)-C(20)-C(19)	120.48(13)	C(42B)-C(43B)-H(43F)	109.5
O(5)-C(20)-C(15)	117.55(13)	H(43D)-C(43B)-H(43F)	109.5
C(19)-C(20)-C(15)	121.97(14)	H(43E)-C(43B)-H(43F)	109.5

Table S2. Torsion angles [°] for **2a**. Symmetry transformations used to generate equivalent atoms.

C(45)-O(12A)-C(46A)-C(48A)	153.9(4)	O(2)-C(4)-C(5)-C(6)	-177.42(13)
C(45)-O(12A)-C(46A)-C(47A)	-100.2(5)	C(3)-C(4)-C(5)-C(6)	3.7(2)
C(45)-O(12B)-C(46B)-C(47B)	-137.8(8)	O(2)-C(4)-C(5)-C(33)	1.1(2)
C(45)-O(12B)-C(46B)-C(48B)	79.8(7)	C(3)-C(4)-C(5)-C(33)	-177.80(13)
C(25)-C(24)-C(23)-C(22)	2.3(2)	C(27)-C(26)-C(25)-O(8)	-179.96(13)
C(28)-C(24)-C(23)-C(22)	-176.74(13)	C(45)-C(26)-C(25)-O(8)	-1.0(2)
C(11)-C(12)-C(13)-O(3)	-179.83(12)	C(27)-C(26)-C(25)-C(24)	1.6(2)
C(37)-C(12)-C(13)-O(3)	-1.0(2)	C(45)-C(26)-C(25)-C(24)	-179.38(14)
C(11)-C(12)-C(13)-C(8)	0.9(2)	C(23)-C(24)-C(25)-O(8)	178.81(13)
C(37)-C(12)-C(13)-C(8)	179.66(13)	C(28)-C(24)-C(25)-O(8)	-2.2(2)
C(13)-C(12)-C(11)-O(4)	178.97(12)	C(23)-C(24)-C(25)-C(26)	-2.9(2)
C(37)-C(12)-C(11)-O(4)	0.16(19)	C(28)-C(24)-C(25)-C(26)	176.14(13)
C(13)-C(12)-C(11)-C(10)	0.5(2)	C(17)-C(16)-C(15)-C(20)	-1.2(2)
C(37)-C(12)-C(11)-C(10)	-178.34(13)	C(17)-C(16)-C(15)-C(14)	178.01(12)
C(2)-C(1)-C(6)-O(1)	179.64(11)	O(5)-C(20)-C(15)-C(16)	-178.84(12)
C(28)-C(1)-C(6)-O(1)	-1.12(19)	C(19)-C(20)-C(15)-C(16)	1.2(2)
C(2)-C(1)-C(6)-C(5)	-0.3(2)	O(5)-C(20)-C(15)-C(14)	2.0(2)
C(28)-C(1)-C(6)-C(5)	178.95(13)	C(19)-C(20)-C(15)-C(14)	-178.03(13)
O(3)-C(13)-C(8)-C(9)	-179.93(12)	C(13)-C(8)-C(9)-C(10)	-1.0(2)
C(12)-C(13)-C(8)-C(9)	-0.6(2)	C(7)-C(8)-C(9)-C(10)	179.64(12)
O(3)-C(13)-C(8)-C(7)	-0.56(19)	C(11)-C(10)-C(9)-C(8)	2.3(2)
C(12)-C(13)-C(8)-C(7)	178.76(12)	C(14)-C(10)-C(9)-C(8)	-177.53(12)
O(4)-C(11)-C(10)-C(9)	179.64(12)	C(9)-C(8)-C(7)-C(3)	84.73(16)
C(12)-C(11)-C(10)-C(9)	-2.0(2)	C(13)-C(8)-C(7)-C(3)	-94.61(15)
O(4)-C(11)-C(10)-C(14)	-0.6(2)	C(9)-C(8)-C(7)-C(30)	-42.31(18)
C(12)-C(11)-C(10)-C(14)	177.84(12)	C(13)-C(8)-C(7)-C(30)	138.35(13)
O(6)-C(18)-C(17)-C(16)	-177.89(12)	C(2)-C(3)-C(7)-C(8)	-88.46(16)
C(19)-C(18)-C(17)-C(16)	0.3(2)	C(4)-C(3)-C(7)-C(8)	92.26(15)
O(6)-C(18)-C(17)-C(21)	2.7(2)	C(2)-C(3)-C(7)-C(30)	38.56(17)
C(19)-C(18)-C(17)-C(21)	-179.10(13)	C(4)-C(3)-C(7)-C(30)	-140.73(14)
C(22)-C(21)-C(17)-C(16)	-86.93(15)	C(16)-C(15)-C(14)-C(10)	87.25(16)
C(32)-C(21)-C(17)-C(16)	40.08(18)	C(20)-C(15)-C(14)-C(10)	-93.60(16)
C(22)-C(21)-C(17)-C(18)	92.43(15)	C(16)-C(15)-C(14)-C(31)	-40.21(18)
C(32)-C(21)-C(17)-C(18)	-140.56(14)	C(20)-C(15)-C(14)-C(31)	138.94(14)

C(18)-C(17)-C(16)-C(15)	0.5(2)	C(9)-C(10)-C(14)-C(15)	-85.06(16)
C(21)-C(17)-C(16)-C(15)	179.86(12)	C(11)-C(10)-C(14)-C(15)	95.15(15)
C(25)-C(26)-C(27)-O(7)	179.78(13)	C(9)-C(10)-C(14)-C(31)	41.61(18)
C(45)-C(26)-C(27)-O(7)	0.8(2)	C(11)-C(10)-C(14)-C(31)	-138.17(14)
C(25)-C(26)-C(27)-C(22)	0.4(2)	C(34)-O(13)-C(33)-C(5)	69.47(17)
C(45)-C(26)-C(27)-C(22)	-178.57(14)	C(6)-C(5)-C(33)-O(13)	66.37(18)
C(2)-C(1)-C(28)-C(24)	85.67(15)	C(4)-C(5)-C(33)-O(13)	-112.16(15)
C(6)-C(1)-C(28)-C(24)	-93.54(15)	C(4)-C(3)-C(2)-C(1)	1.3(2)
C(2)-C(1)-C(28)-C(29)	-40.84(17)	C(7)-C(3)-C(2)-C(1)	-178.04(12)
C(6)-C(1)-C(28)-C(29)	139.95(13)	C(6)-C(1)-C(2)-C(3)	0.5(2)
C(23)-C(24)-C(28)-C(1)	-88.65(15)	C(28)-C(1)-C(2)-C(3)	-178.69(13)
C(25)-C(24)-C(28)-C(1)	92.38(16)	C(46B)-O(12B)-C(45)-C(26)	-171.0(4)
C(23)-C(24)-C(28)-C(29)	37.85(18)	C(46A)-O(12A)-C(45)-C(26)	179.9(3)
C(25)-C(24)-C(28)-C(29)	-141.12(14)	C(27)-C(26)-C(45)-O(12B)	-11.3(4)
C(24)-C(23)-C(22)-C(27)	-0.4(2)	C(25)-C(26)-C(45)-O(12B)	169.7(3)
C(24)-C(23)-C(22)-C(21)	-179.77(13)	C(27)-C(26)-C(45)-O(12A)	-40.3(3)
O(7)-C(27)-C(22)-C(23)	179.60(13)	C(25)-C(26)-C(45)-O(12A)	140.7(2)
C(26)-C(27)-C(22)-C(23)	-1.0(2)	C(18)-C(19)-C(41)-O(11B)	-164.2(5)
O(7)-C(27)-C(22)-C(21)	-1.0(2)	C(20)-C(19)-C(41)-O(11B)	15.7(5)
C(26)-C(27)-C(22)-C(21)	178.40(13)	C(18)-C(19)-C(41)-O(11A)	-134.39(16)
C(17)-C(21)-C(22)-C(23)	87.46(16)	C(20)-C(19)-C(41)-O(11A)	45.4(2)
C(32)-C(21)-C(22)-C(23)	-39.63(19)	C(38B)-O(10)-C(37)-C(12)	88.9(3)
C(17)-C(21)-C(22)-C(27)	-91.90(16)	C(38A)-O(10)-C(37)-C(12)	175.6(2)
C(32)-C(21)-C(22)-C(27)	141.00(14)	C(13)-C(12)-C(37)-O(10)	40.13(19)
C(2)-C(3)-C(4)-O(2)	177.75(13)	C(11)-C(12)-C(37)-O(10)	-141.10(14)
C(7)-C(3)-C(4)-O(2)	-2.9(2)	C(33)-O(13)-C(34)-C(36)	-155.77(14)
C(2)-C(3)-C(4)-C(5)	-3.4(2)	C(33)-O(13)-C(34)-C(35)	80.61(18)
C(7)-C(3)-C(4)-C(5)	175.91(12)	C(101)-O(88)-C(100)-O(12)	-0.3(3)
O(6)-C(18)-C(19)-C(20)	177.99(12)	C(101)-O(88)-C(100)-C(99)	178.32(17)
C(17)-C(18)-C(19)-C(20)	-0.3(2)	C(100)-O(88)-C(101)-C(102)	173.81(17)
O(6)-C(18)-C(19)-C(41)	-2.2(2)	C(37)-O(10)-C(38A)-C(40A)	175.7(3)
C(17)-C(18)-C(19)-C(41)	179.55(14)	C(37)-O(10)-C(38A)-C(39A)	-55.3(3)
O(5)-C(20)-C(19)-C(18)	179.53(13)	C(37)-O(10)-C(38B)-C(40B)	47.3(6)
C(15)-C(20)-C(19)-C(18)	-0.5(2)	C(37)-O(10)-C(38B)-C(39B)	176.8(4)
O(5)-C(20)-C(19)-C(41)	-0.3(2)	C(19)-C(41)-O(11A)-C(42A)	173.9(2)
C(15)-C(20)-C(19)-C(41)	179.70(14)	C(41)-O(11A)-C(42A)-C(44A)	77.4(3)
O(1)-C(6)-C(5)-C(4)	178.31(13)	C(41)-O(11A)-C(42A)-C(43A)	-162.0(3)
C(1)-C(6)-C(5)-C(4)	-1.8(2)	C(19)-C(41)-O(11B)-C(42B)	-170.5(13)
O(1)-C(6)-C(5)-C(33)	-0.2(2)	C(41)-O(11B)-C(42B)-C(43B)	-167.2(15)
C(1)-C(6)-C(5)-C(33)	179.68(13)	C(41)-O(11B)-C(42B)-C(44B)	103.6(12)
